# Di‐(2‐ethylhexyl) phthalate exposure induces female reproductive toxicity and alters the intestinal microbiota community structure and fecal metabolite profile in mice

**DOI:** 10.1002/tox.23121

**Published:** 2021-03-04

**Authors:** Xufeng Fu, Hang Han, Yuanyuan Li, Bo Xu, Wenjie Dai, Yaoxu Zhang, Feng Zhou, Huiming Ma, Xiuying Pei

**Affiliations:** ^1^ Key Laboratory of Fertility Preservation and Maintenance of Ministry of Education Ningxia Medical University Yinchuan China; ^2^ State Key Laboratory of Environmental Chemistry and Ecotoxicology, Research Center for Eco‐Environmental Sciences Chinese Academy of Sciences Beijing China

**Keywords:** DEHP, female reproductive toxicity, intestinal microbiota, metabolite profile

## Abstract

Di‐(2‐ethylhexyl) phthalate (DEHP) is one of the most commonly used plasticizers, and it is widely applied in various plastic products. DEHP is an endocrine‐disrupting chemical (EDC) that has been shown to disrupt the function of reproductive system in females. Although many studies have shown that DEHP potentially causes female reproductive toxicity, including depletion of the primordial follicle and decreased sex hormone production, the specific mechanisms by which DEHP affects female reproduction remain unknown. In recent years, research focused on the intestinal flora has provided an idea to eliminate our confusion, and gut bacterial dysbiosis may contribute to female reproductive toxicity. In the present study, the feces of DEHP‐exposed mice were collected and analyzed using 16S rRNA amplicon sequencing and untargeted global metabolite profiling of metabolomics. DEHP obviously causes reproductive toxicity, including the ovarian organ coefficient, estradiol level, histological features of the ovary and estrus. Furthermore, DEHP exposure alters the structure of the intestinal microbiota community and fecal metabolite profile in mice, suggesting that the reproductive toxicity may be caused by gut bacterial dysbiosis and altered metabolites, such as changes in the levels of short‐chain fatty acid (SCFA). Additionally, it is well known that changes in gut microbiota and fecal metabolites cause inflammation and tissue oxidative stress, expectedly, we found oxidative stress in the ovary and systemic inflammation in DEHP exposed mice. Thus, based on our findings, DEHP exposure may cause gut bacterial dysbiosis and altered metabolite profiles, particularly SCFA profiles, leading to oxidative stress in the ovary and systemic inflammation to ultimately induce female reproductive toxicity.

## 1 INTRODUCTION

Di‐(2‐ethylhexyl) phthalate (DEHP) is one of the most commonly used plasticizers, and it is widely used in the production of various cosmetics, personal care products, food storage containers, pharmaceuticals, building materials, children's toys, medical tubing, and other polyvinyl chloride products.^1^, ^2^, ^3^ DEHP is continuously released from plastic products and directly infiltrates food, water and air; thus, humans are exposed to DEHP daily via ingestion, inhalation, and dermal absorption.^2^, ^4^, ^5^, ^6^ In recent years, DEHP and its active metabolic product, mono(2‐ethylhexyl) phthalate (MEHP), have been successively detected in many tissues, including the liver, blood, cord blood, breast milk, placenta, amniotic fluid, and early gestation villi.^7^, ^8^, ^9^ According to previous studies, DEHP is associated with hepatic, renal and neural injures or diseases.^10^, ^11^, ^12^ In addition, DEHP is an endocrine‐disrupting chemical (EDC) that has been shown to disrupt the function of reproductive system in both females and males.^13^, ^14^ Several studies have confirmed that the obvious toxicity of DEHP is mediated by its effects on gonadal steroidogenesis and an accompanying decrease in reproductive function and fertility.^13^, ^15^, ^16^ The average exposure of DEHP in humans ranges from 3 to 30 mg/kg (body weight)/d, and occupational exposure levels have been calculated to even reach up to 300 to 600 mg/kg/d.^17^, ^18^ Based on epidemiological data, decreased conception rates, increased miscarriage rates, decreased estrogen levels, and abnormal ovulation are associated with the occupational exposure of female workers to DEHP.^19^, ^20^ Thus, the health risks of DEHP exposure in humans have attracted increasing attention.

The ovary is the primary reproductive organ in the female, and it regulates female endocrinology and provides a microenvironment for follicle development.^21^ DEHP and MEHP have been shown to induce a depletion of the primordial follicles and a decrease in sex hormone production.^22^, ^23^, ^24^ However, the precise mechanisms by which DEHP affects female reproduction remain unclear. The human gastrointestinal tract contains 10^13^–10^14^ microbiota that consist of 1500 microbial species and are characterized by more than 3 million genotypes, and numerous studies have shown that the intestinal microbiota is inextricably linked to human health.^25^, ^26^ Approximately 1000 bacterial species and 7000 bacterial strains have been identified using the currently available advanced sequencing technology.^27^ Recent studies have revealed the bidirectional relationship between the estrogen level and gut microbiota in females with diseases induced by abnormal estrogen levels; estrogens are potentially regulated by the gut microbiota through secretion of β‐glucuronidase, which deconjugates estrogens into active forms, and estrogens regulate the gut microbiota through immunoregulation.^28^, ^29^, ^30^ In addition, gut microbiota dysbiosis may increase intestinal permeability and alter the levels of host gut metabolites, allowing bacterial endotoxins, such as lipopolysaccharide, to be transported into the circulation and activate the inflammatory response to promote disease development in females.^31^, ^32^ Therefore, we hypothesized that women of child‐bearing age who are exposed to DEHP would exhibit abnormal follicular development and infertility that may be mediated by alterations in the gut bacterial composition and metabolite profiles.

In the present study, we aimed to determine the effects of DEHP exposure on ovarian damage and homeostasis of the gut microbiome and fecal metabolome, and to explore the relationship between DEHP‐induced reproductive disease and the gut microbiome or fecal metabolome in an effort to provide deeper insights into the etiology of reproductive toxicity. For the precise analysis, mice were exposed to different concentrations of DEHP for 30 days, and then the effects of DEHP exposure on reproductive toxicity, including ovarian pathology and serum hormone levels, were detected, and fecal microbiome‐metabolome responses were evaluated using 16S rRNA pyrosequencing and nontargeted metabolomics. Together with the dual omics approach, we attempted to identify differences in strains and metabolites between DEHP‐exposed and normal mice, as the differences in strains and metabolites can be used as biomarkers for the diagnosis or auxiliary diagnosis of DEHP‐induced reproductive toxicity. More importantly, our study will more accurately describe the intestinal microbial and fecal metabolic states associated with reproductive toxicity and enable a complete understanding of the mechanisms of the gut microbiota and host health that may facilitate further studies and provide a valuable reference for human fecal microbiota transplantation to treat metabolic reproductive diseases in the future.

## 2 MATERIALS AND METHODS

### 2.1 Animals

All procedures were performed according to the regulations of the Ethics Committee of Ningxia Medical University, Ningxia, China. Four‐week‐old ICR mice were provided by the animal care facility of Ningxia Medical University. Animals were housed identically and maintained in a controlled room at a temperature of 21–22°C on a 12 h dark/12 h light cycle with free access to food and water. All animal experiments were conducted in accordance with the National Institutes of Health Guide and with the approval of the Ningxia Medical University Committee for the Care and Use of Laboratory Animals (NXMU‐).

### 2.2 DEHP exposure and sample collection

DEHP (99% purity) was purchased from Sigma‐Aldrich, and diluted in edible corn oil (Jinlong, China) to prepare stock and working solutions. The doses administered in this study were 500 and 1500 mg/kg body weight per day. The doses were primarily selected based on the equivalent dose ratio calculated using the surface areas of humans and mice. Thirty female mice were randomly and divided into three groups, and animals in the control group were administered an equal volume of corn oil by oral gavage. All animals were intragastrically administered DEHP (mixed with corn oil) for 30 days and doses were adjusted daily according to changes in body weight. The doses of DEHP were based on previous studies.^14^, ^33^, ^34^ 30 days later, mice in each group was anesthetized by intraperitoneally injecting 0.3 mL/100 g of body weight of a 10% chloral hydrate (Sigma, Missouri) solution, and the ovaries and venous blood were collected. The ovarian organ coefficient was evaluated by the ratio of two ovary weight to body weight.

### 2.3 Analysis of estrous cyclicity

During DEHP exposure, vaginal smears were collected from the mice each day to monitor estrous cyclicity. The stage of estrus was determined by performing vaginal cytology using a light microscope and was recorded based on previously defined and well‐documented criteria.^35^ The percentage of days in estrus was calculated by dividing the number of days in estrus by the number of days in the study and multiplying that value by 100. The percentage of days in metestrus/diestrus was calculated by dividing the number of days in either metestrus or diestrus by the number of days in the study and multiplying that value by 100. The evaluation of estrous cyclicity was repeated 3 times.

### 2.4 Hematoxylin and eosin staining of ovarian tissues

Ovarian tissue samples were collected from the euthanized mice, fixed with 4% paraformaldehyde for 24 h, and subsequently processed for histological staining. Tissues were then dehydrated using increasing concentrations of alcohol ranging from 60% to absolute alcohol, cleared and infiltrated with xylene and embedded in paraffin. The paraffin blocks of ovaries were sectioned into 5‐mm slices and sequentially stained with a hematoxylin solution for 5 min and an eosin solution for 5 min. All reagents were purchased from Solarbio Science and Technology Ltd. (Beijing, China). The slides were viewed and images were captured under an Olympus microscope and camera system. The whole ovary was continuously sliced into numerous sections that were placed on glass slides in order, and the numbers of whole follicles and atretic follicles were observed under a microscope and recorded from every five sections to compare the numbers of atretic follicles between groups.

### 2.5 Measurement of estradiol levels

Blood was collected from euthanized mice and plasma was separated by centrifugation at 3000 rpm for 15 min within 1 h after blood collection and stored at −80°C until analysis. Estradiol levels were measured in plasma samples using an enzyme‐linked immunosorbent assay (ELISA) kit (Beyond Biotechnology, Shanghai, China) according to the manufacturer's instructions. Briefly, the estradiol standard and the plasma samples were added to the 96‐well plate provided with the kit, horseradish peroxidase‐labeled estradiol was added to the wells containing the samples to competitively bind the estradiol antibody labeled on the plate and then the free estradiol was removed by washing the plate. After coloration and termination, the absorbance of each well in the plate was detected using a multimode microplate reader (Thermo Scientific) at 450 nm. Finally, the estradiol levels in the samples were calculated from the standard curve.

### 2.6 Fecal sample collection and DNA extraction

Fresh fecal samples were collected from control, 500 and 1500 mg/kg (fecal samples from random six animals in each group) DEHP‐exposed groups, placed in sterile tubes in an ice bath, and immediately transferred to the laboratory. All fecal samples were stored at −80°C. Microbial genomic DNA was isolated and purified from each fecal sample using a DNA Extraction Kit (Omega Bio‐tek, Norcross) according to the manufacturer's instruction. The DNA integrity and concentrations were determined using a Nanodrop2000 spectrophotometer (Thermo Fisher Scientific) and Qubit R 2.0 Fluorometer (Life Technologies, CA).

### 2.7 16S rDNA sequencing and bioinformatics analysis

Polymerase chain reaction (PCR) was used to amplify the V3‐V4 region of the 16S rDNA with specific primers. Equimolar concentrations of PCR products were purified, quantified and sequenced using the Illumina Hiseq2500 platform according to the standard protocols provided by Novogene Technology Co. Ltd. (Beijing, China). Paired‐end raw reads were merged from the original DNA fragments with FLASH (version 1.2.7). After qualified filtering with Trimmomatic (version 0.33), clean reads were extracted and effective tags were produced by removing the chimeric sequences using UCHIME (version 4.2). Finally, high‐quality effective tags were evaluated according to Q20 and Q30 bases and used for bioinformatics analysis.

Using Uparse, all effective reads were clustered into operational taxonomic units (OTUs) based on 97% sequence similarity using QIIME (version 1.8.0), and the representative sequence of each OTU was taxonomically classified based on the Silva database. These OTUs were used for taxonomic annotation by aligning them with bacterial, fungal, Archaea, and virus sequences from the NT (Version: 2014) database of NCBI using the BLAST algorithm (evalue ≤1e‐5). Differences in the abundance of various taxa and the microbial evolutionary relationships were evaluated using the QIIME and MEGAN software packages, respectively. The species diversity was determined and the ACE, Chao1 estimators, Simpson and Shannon indices were calculated using Qiime. The *β*‐diversity of the microbial communities was explored using principal coordinate analysis (PCoA) plots and nonmetric multidimensional scaling (NMDS) based on weighted UniFrac distances. The linear discriminant analysis effect size (LEfSe) method was used to identify the genomic features and analyze the biomarkers of the groups.^36^, ^37^

### 2.8 Fecal metabolomics profiling and data analysis

Untargeted global metabolite profiling of fecal samples was performed using an ultraperformance liquid chromatography/quadrupole orthogonal acceleration time of flight tandem mass spectrometer (UPLC/Q‐TOF MS) due to its high sensitivity, acquisition rates, and capability of collecting accurate mass data. Briefly, 1.0 mL of an extraction solution composed of methanol/acetonitrile/deionized‐water (2:2:1, v/v/v) and containing the internal standard (L‐2‐chlorophenylalanine, 2 μg/mL) was added to each fecal sample (50 mg), which was homogenized by ultrasound and then sonicated in ice water bath. After centrifugation, the supernatant was collected in a glass vial and dried in a vacuum concentrator at 37°C. Then, the dried samples were reconstituted in 50% acetonitrile, sonicated, and centrifuged, and 75 μL of the supernatant were transferred to a fresh glass vial for the LC/MS analysis. The quality control (QC) sample was prepared by mixing an equal volume of the supernatants from all of the samples. UHPLC separation was conducted using a 1290 Infinity series UHPLC System (Agilent Technologies) equipped with a UPLC BEH Amide column (2.1 × 100 mm, 1.7 μm, Waters). A TripleTOF 6600 mass spectrometer (AB Sciex) was used to acquire MS/MS spectra on an information‐dependent basis (IDA) during an LC/MS experiment. For metabolite identification, the raw data (.wiff) files were converted to the mzXML format by ProteoWizard, and the peak deconvolution, alignment and integration were processed using the R package XCMS (version 3.2). Then, the metabolites were identified based on the original secondary MS mass database of Biotree Technology Co. Ltd. (Shanghai, China).

The principal components of the metabolites with variable important projection (VIP) values >1.0 and *p* value <.05 in Student's t test were considered significantly different between the groups. The principal component analysis (PCA) and orthogonal partial least squares discriminant analysis (OPLS‐DA) were performed to visualize metabolic differences among the groups. The identified metabolites were validated by searching the Kyoto Encyclopedia of Genes and Genomes (KEGG) database, and each significantly different metabolite was cross‐referenced with KEGG pathways and with the free web‐based tool MetaboAnalyst (http://www.metaboanalyst.ca) for pathway topology.

### 2.9 Measurement of SOD and MDA levels

The levels of superoxide dismutase (SOD) in the ovaries of the 500 and 1500 mg/kg DEHP‐exposed and the control groups were quantified using a Total Superoxide Dismutase Assay Kit with NBT (Beyotime, Shanghai, China) according to the manufacturer's instructions. Superoxide anion radicals that are not scavenged by SOD reduce NBT to a blue formazan product. The absorbance of the blue formazan product in each sample was detected with a multimode microplate reader at 450 nm. Malondialdehyde (MDA) levels in ovarian tissue extract were measured using a Lipid Peroxidation MDA Assay Kit (Beyotime, Shanghai, China), which is based on the ability of MDA to react with thiobarbituric acid to generate a red product. The absorbance of each sample was determined using a multimode microplate reader (Thermo Scientific) at 535 nm.

### 2.10 Measurement of proinflammatory factors

IL‐1β and TNF‐α were measured in plasma samples using an enzyme‐linked immunosorbent assay (ELISA) kit (Jonln Biotech., Shanghai, China) according to the manufacturer's instructions. Briefly, the standard of IL‐1β, TNF‐α and the plasma samples were added to the 96‐well plate provided with the kits, horseradish peroxidase‐labeled IL‐1β and TNF‐α were added to the wells containing the samples to competitively bind the IL‐1β and TNF‐α antibody labeled on the plate and then the free IL‐1β and TNF‐α were removed by washing the plate. After coloration and termination, the absorbance of each well in the plate was detected using a multimode microplate reader (Thermo Scientific) at 450 nm. Finally, the levels of IL‐1β and TNF‐α in the samples were calculated from the standard curve.

### 2.11 Statistical Analysis

The differences among groups are presented as means ± SD and were analyzed using one‐way ANOVA with GraphPad Prism 5.0 software. If the variance was equal, the Student–Newman–Keuls (SNK) test was used for the pairwise comparison; otherwise, the Games‐Howell test was used. *p* < .05 was considered a significant difference in all analyses. ANOVA was performed to identify significant differences in the abundances of intestinal bacteria and metabolites. Pearson's correlation coefficients were calculated to recognize the correlations between perturbed intestinal microbiota and changes in fecal metabolites.

## 3 RESULTS

### 3.1 Reproductive toxicity was induced by DEHP exposure

**FIGURE 1:**
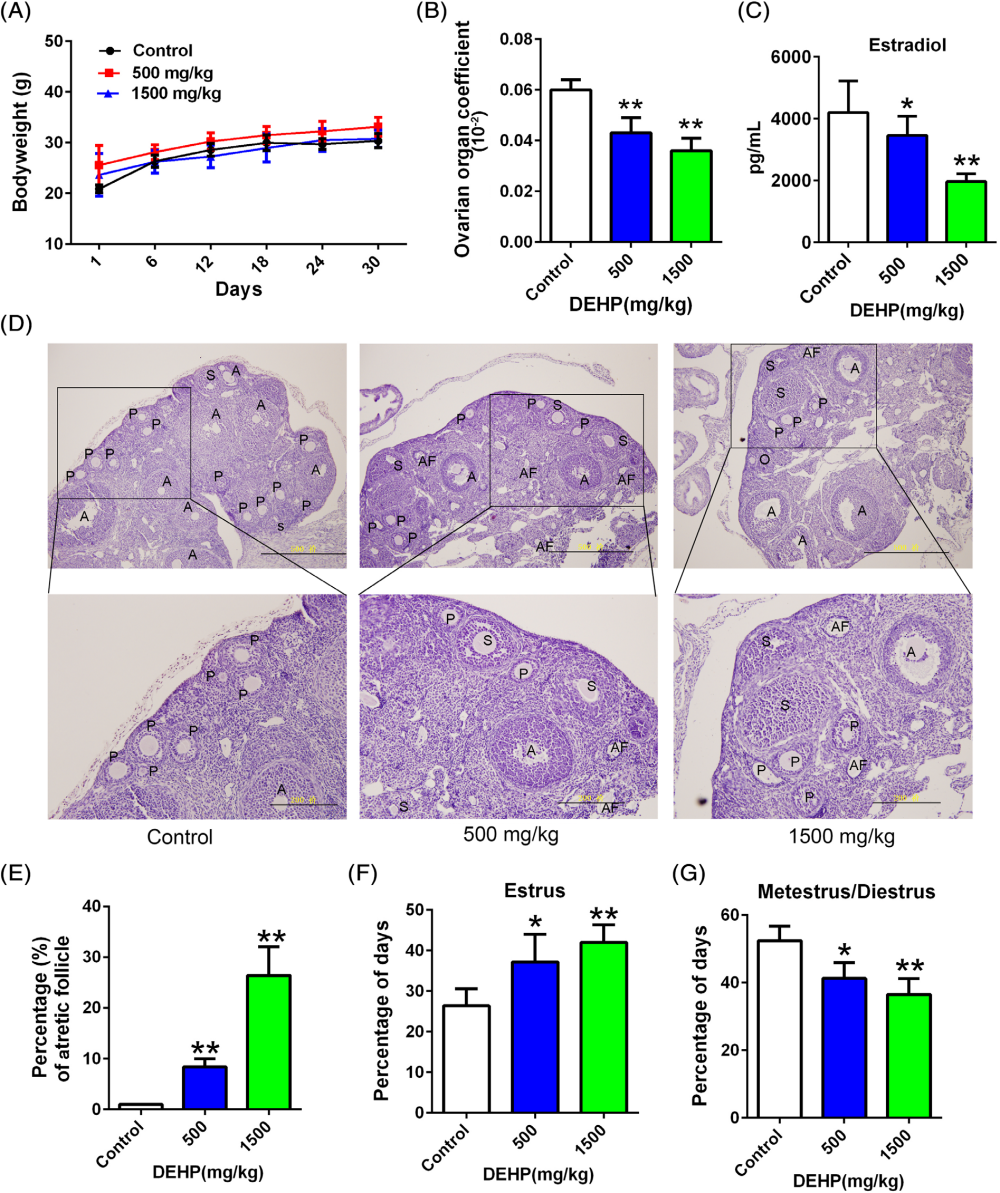
The characteristics of the female reproductive system were changed after DEHP exposure. (A) The body weight was not changed after DEHP exposure. (B) The ovarian organ coefficient was altered following DEHP exposure. (C) Changes in the plasma estradiol levels after DEHP exposure. (D) Changes in the histological features of the ovary after DEHP exposure; scale bar: upper row is 500 μm and under is 200 μm; primary (P), secondary (S), antral follicles (A), oocytes (O), and atretic follicles (AF). (E) The number of atretic follicles was increased after DEHP exposure. Estrus (F) and metestrus/diestrus (G) were significantly altered by DEHP. *p < .05 and **p < .01 for the comparison of the DEHP‐exposed groups with the control group [Color figure can be viewed at wileyonlinelibrary.com]

During the 30 days of DEHP exposure, the control, 500 and 1500 mg/kg DEHP groups exhibited similar levels of body weight gain, and thus continuous DEHP exposure for 30 days did not alter the body weight of mice (Figure 1(A)). Significantly decreased ovary organ coefficient (Figure 1(B)) and estradiol levels (Figure 1(C)) were also observed in all DEHP‐treated groups. Significant differences in follicle morphology were observed in histological examinations of the ovarian tissues from mice exposed to DEHP under a light microscope. The numbers of primary, secondary and antral follicles were decreased, and the numbers of oocytes in the primary follicles were obviously decreased by the 500 and 1500 mg/kg DEHP treatments. In the groups treated with DEHP, oocyte loss occurred in the primary follicles, along with a loose structure and detachment of granular cells, wide intercellular spaces between granulosa cells and theca cells (Figure 1(D)), and an increase in the number of atretic follicles (Figure 1(E)). Estrus was increase and metestrus/diestrus was decrease by DEHP exposure (Figure 1(F) and (G)). Based on these results, DEHP obviously causes female reproductive toxicity.

### 3.2 Gut microbiota dysbiosis was induced by DEHP exposure

The mouse fecal microbiota was characterized by performing 16S rDNA sequencing. Alpha diversity was used as a measure of the complexity of the species diversity and richness by calculating several indices, including Chao1, and Shannon and Simpson indices. The Chao1 histogram showed the species number determined in each sample. The flattening of the rarefaction curve based on the values of OTUs and Shannon indices indicated that our data volume covered all species of the community in the samples. Moreover, we revealed both increases and decreases in different gut bacterial species in the control, 500 and 1500 mg/kg DEHP‐exposed groups (Figure S1), respectively.

**FIGURE 2:**
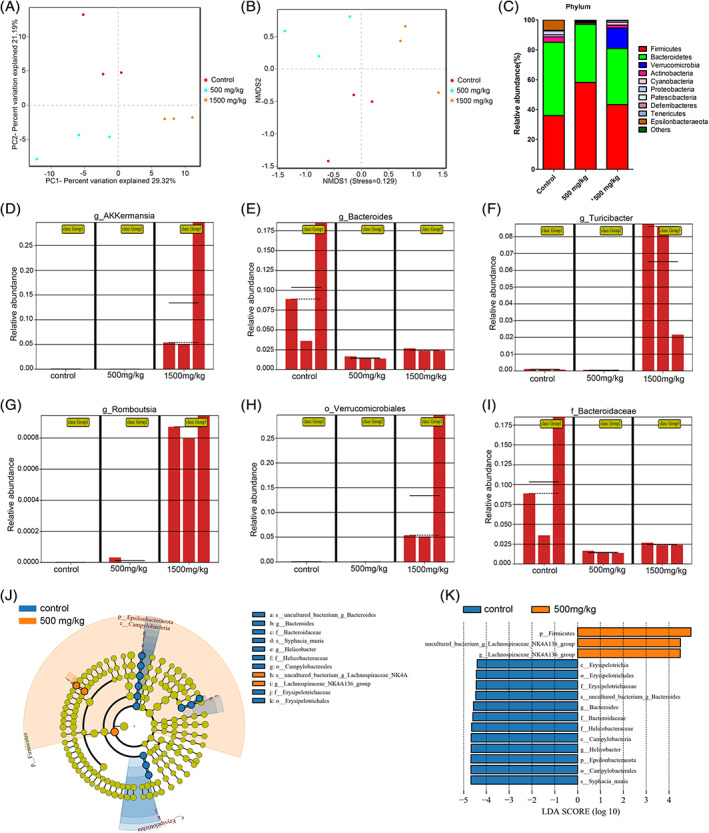
Comparison of the fecal microbiota structures and distributions among control, 500 and 1500 mg/kg DEHP exposed groups. (A) PCoA analysis of the control, 500 and 1500 mg/kg DEHP groups. (B) NMDS analysis of the control, 500 and 1500 mg/kg DEHP groups. (C) Relative abundance of the fecal microbiota at the phylum level. The relative abundance of g_*Akkermansia* (D), g_*Bacteroides* (E), g_*Turicibacter* (F), g_*Romboutsia* (G), o_Verrucomicrobiales (H), and f_Bacteroidaceae (I) after DEHP exposure. Taxa are represented as g (genus), o (order), and f (family). (J) and (K) are obtained from the LEfSe analysis. (J) The taxonomic cladograms of the control and 500, the blue and orange dots are proportional to the degree of enrichment of certain taxa between two comparative groups. (K) Taxa with LDA scores greater than 4 according the comparison of control and the 500 mg/kg‐DEHP exposed group. Taxa are represented as c (class), o (order), f (family), and g (genus) [Color figure can be viewed at wileyonlinelibrary.com]

By examining the unweighted UniFrac distance, 500 and 1500 mg/kg DEHP‐exposed mice and controls were separated on the PCoA plot (Figure 2(A)) and NMDS (Figure 2(B)). PCoA and NMDS analyses of the relative abundance of different bacterial taxa indicated a considerable separation among these three groups, suggesting a change in the structure of the bacterial community in DEHP‐exposed mice. The gut microbiota in the control, 500 and 1500 mg/kg DEHP‐exposed groups were dominated by five phyla: *Firmicutes*, *Bacteroidetes*, *Verrucomicrobia*, *Actinobacteria*, and *Epsilonbacteraeota* (Figure 2(C)). The relative abundance of *Firmicutes* was increased, and the relative abundances of *Bacteroidetes*, *Actinobacteria*, and *Epsilonbacteraeota* were decreased in the DEHP‐exposed groups compared to the control group. Interestingly, a higher abundance of *Verrucomicrobia* was detected and this phylum was an important component of the bacterial community in 1500 mg/kg DEHP‐exposed group (Figure 2(C)). To further identify the specific bacterial species as the biomarkers after DEHP exposing, the abundances *Akkermansia* (Figure 2(D)), *Turicibacter* (Figure 2(F)), *Romboutsia* (Figure 2(G)) and Verrucomicrobiales (Figure 2(H)) were increased in mice exposed to 1500 mg/kg DEHP and the abundances of *Bacteroides* (Figure 2(E)) and *Bacteroidaceae* (Figure 2(I)) were decreased in mice exposed to 500 and 1500 mg/kg DEHP. The taxonomic distributions of the fecal microbiota showed significant differences at the class, family, and genus levels in mice exposed to 500 and 1500 mg/kg DEHP, as shown in Figure S2.

A linear discriminant analysis (LDA) effect size (LEfSe) analysis was performed on pairwise comparisons to further characterize the phylotypes of the gut microbiota distinguishing the three groups, and the cladogram shows the primary bacteria identified in the fecal microbiota of the control, 500 and 1500 mg/kg DEHP‐exposed groups. As shown in the cladogram, significant differences in the taxa were evident among the three groups. According to the LEfSe analysis and LDA score (LDA score ≥ 4), higher abundances were observed for 12 taxa (*Syphacia muris*, Campylobacterales, Epsilonbacteraeota, *Helicobacter*, *Campylobacter*, Helicobacteraceae, Bacteroidaceae, *Bacteroides*, uncultured *Bacteroides* bacterium, *Erysipelotrichaceae*, *Erysipelotrichales* and *Erysipelotrichia*) in the control group and 3 taxa (*Firmicutes*, uncultured bacterium in the *Lachnospiraceae*_NK4A136_group and *Lachnospiraceae*_NK4A136_group) in 500 mg/kg DEHP‐exposed group (Figure 2(J) and (K)), as well as 9 taxa (*Helicobacteraceae*, *Campylobacterales*, *Epsilonbacteraeota*, *Syphacia muris*, *Helicobacter*, *Campylobacter*, *Bacteroides, Bacteroidaceae* and uncultured *Bacteroides* bacterium) in the control group and 9 taxa (*Peptostreptococcaceae*, *Turicibacter*, uncultured *Turicibacter* bacterium, Verrucomicrobiales, uncultured *Akkermansia* bacterium, *Verrucomicrobia*, *Akkermansiaceae*, *Akkermansia* and *Verrucomicrobia*) in 1500 mg/kg DEHP‐exposed group (Figure S3A and 3B). Higher abundances of 11 taxa (*Verrucomicrobia*, *Akkermansiaceae*, uncultured *Akkermansia* bacterium, *Akkermansia*, *Verrucomicrobia*, *Verrucomicrobiales*, *Erysipelotrichia*, *Erysipelotrichaceae*, *Turicibacter*, *Erysipelotrichales* and uncultured *Turicibacter* bacterium) in the 1500 mg/kg DEHP‐exposed group and 4 taxa (uncultured *Roseburia* bacterium, *Roseburia*, uncultured bacterium in the *Lachnospiraceae*_NK4A136_group and *Lachnospiraceae*_NK4A136_group) in the 500 mg/kg DEHP‐exposed group were observed (Figure S3C and S3D). These results revealed a significant difference in the gut microbiota between the control and DEHP‐exposed groups. Here, we particularly considered obvious differences in the gut microbiota after DEHP exposure.

**FIGURE 3:**
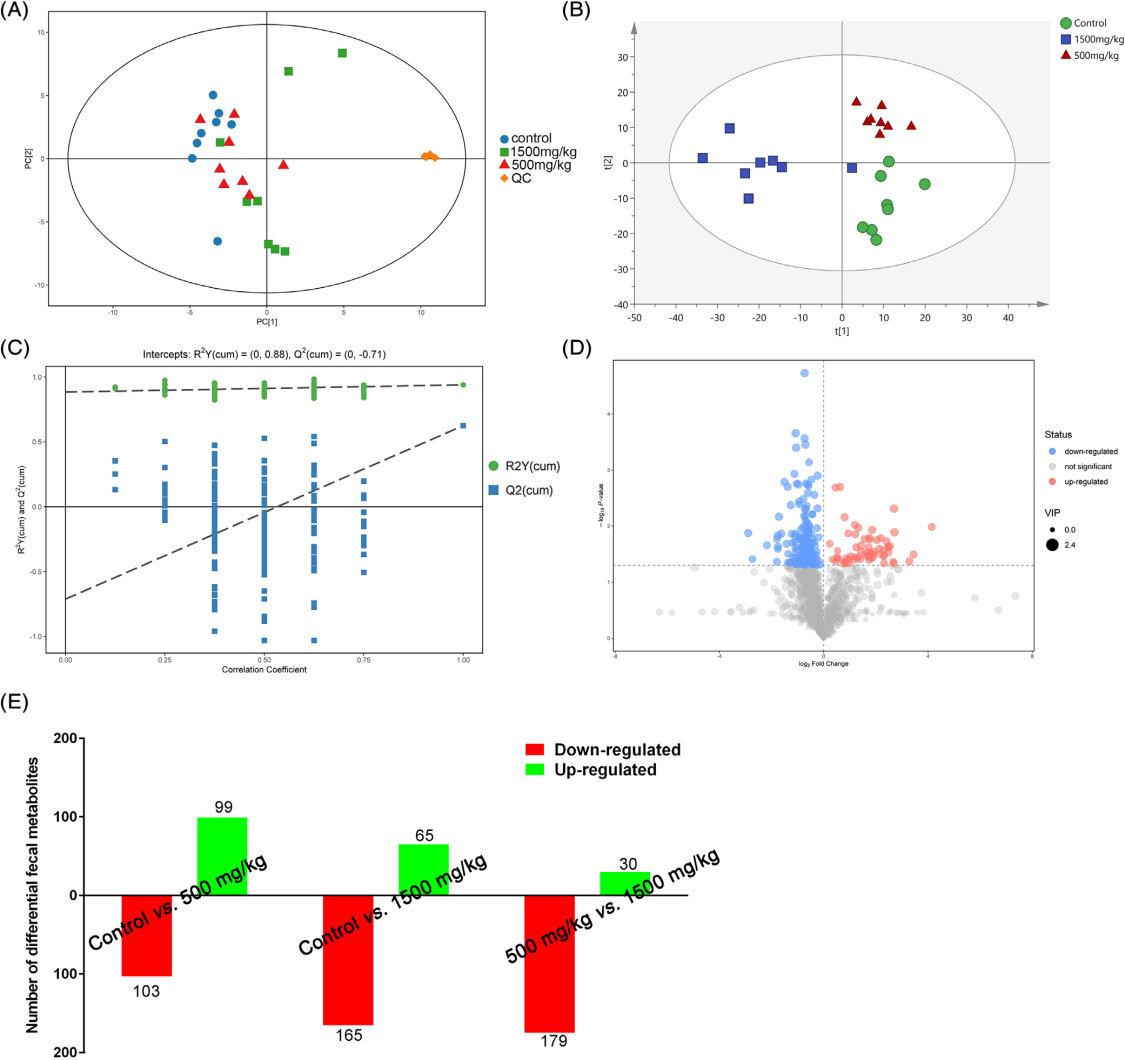
Metabolic profiles of the fecal samples from the control, 500 and 1500 mg/kg DEHP‐exposed groups. (A) PCA score plots of fecal samples derived from the metabolite profiles, QC: quality control, (n = 8). (B) OPLS‐DA score scatter plots of fecal samples derived from the metabolite profiles (n = 8). (C) Permutation test of the OPLS‐DA model for the control and 1500 mg/kg DEHP groups. (D) Volcano plot of the control and 1500 mg/kg DEHP‐exposed groups, where each point represents a metabolite. Red dots represent up‐regulated metabolites, blue dots represent down‐regulated metabolites, and gray dots indicate nonsignificant differences. (E) The number of differential fecal metabolites after 500 and 1500 mg/kg DEHP exposure compared to control. Red represents down‐regulated and green represents up‐regulated [Color figure can be viewed at wileyonlinelibrary.com]

### 3.3 Effects of DEHP exposure on fecal metabolite profiles in mice

Using an untargeted strategy, the fecal metabolome associated with functional characteristics of the gut microbiome was studied. A total of 1656 peaks was ultimately identified and quantified using the UHPLC‐QTOFMS analysis, and 1609 peaks and 652 metabolites remained after eliminating noise using the interquartile range denoising method. These compounds were annotated based on a comparison with reference compounds in internal libraries or authentic reference standards. These metabolites included amino acids, carbohydrates, fatty acids, amines, polyols, organic acids, and nucleotides involved in multiple biochemical processes in mice. A principal component analysis (PCA) and univariate statistical analysis were performed to detect and visualize the trends and outliers in the fecal metabolome, and the result showed the data distribution and the separation in the fecal metabolic structures of the three groups, which were stable and reliable (Figure 3(A)). Additionally, DEHP exposure exerted a substantial effect on modulating the fecal metabolome composition. An orthogonal projections to latent structures discriminant analysis (OPLS‐DA) was performed to obtain a better understanding of the effect of DEHP exposure on the classification, and the result indicated the stable and accurate prediction of the current models (Figure 3(B)). A random permutations test was performed to verify the validity and robustness of the OPLS‐DA model, and a negative corresponding Q2 value was considered valid and at low risk of over fitting. As shown in Figure 3(C), the comparison of the control and 1500 mg/kg DEHP‐exposed group was valid, and comparisons between the control and 500 mg/kg DEHP group (Figure S4A) and between the 500 and 1500 mg/kg DEHP groups were also valid (Figure S4B). Significantly altered metabolites were identified based on the following criteria: variable importance of the projection (VIP) values >1.0 calculated using the OPLS‐DA model and *p* values <.05 calculated using Student's t test. In the comparison of the control and 1500 mg/kg DEHP‐exposed groups, 165 metabolites were downregulated and 65 were upregulated, which were visualized in a volcano plot in Figure 3(D). In the comparison of the control and 500 mg/kg DEHP‐exposed groups, 103 metabolites were downregulated and 99 were upregulated (Figure S4C). In the comparison of the 500 and 1500 mg/kg DEHP‐exposed groups, 179 metabolites were downregulated and 30 were upregulated (Figure S4D). The numbers of differentially altered metabolites in the control, 500 and 1500 mg/kg DEHP groups are presented in Figure 3E.

**FIGURE 4:**
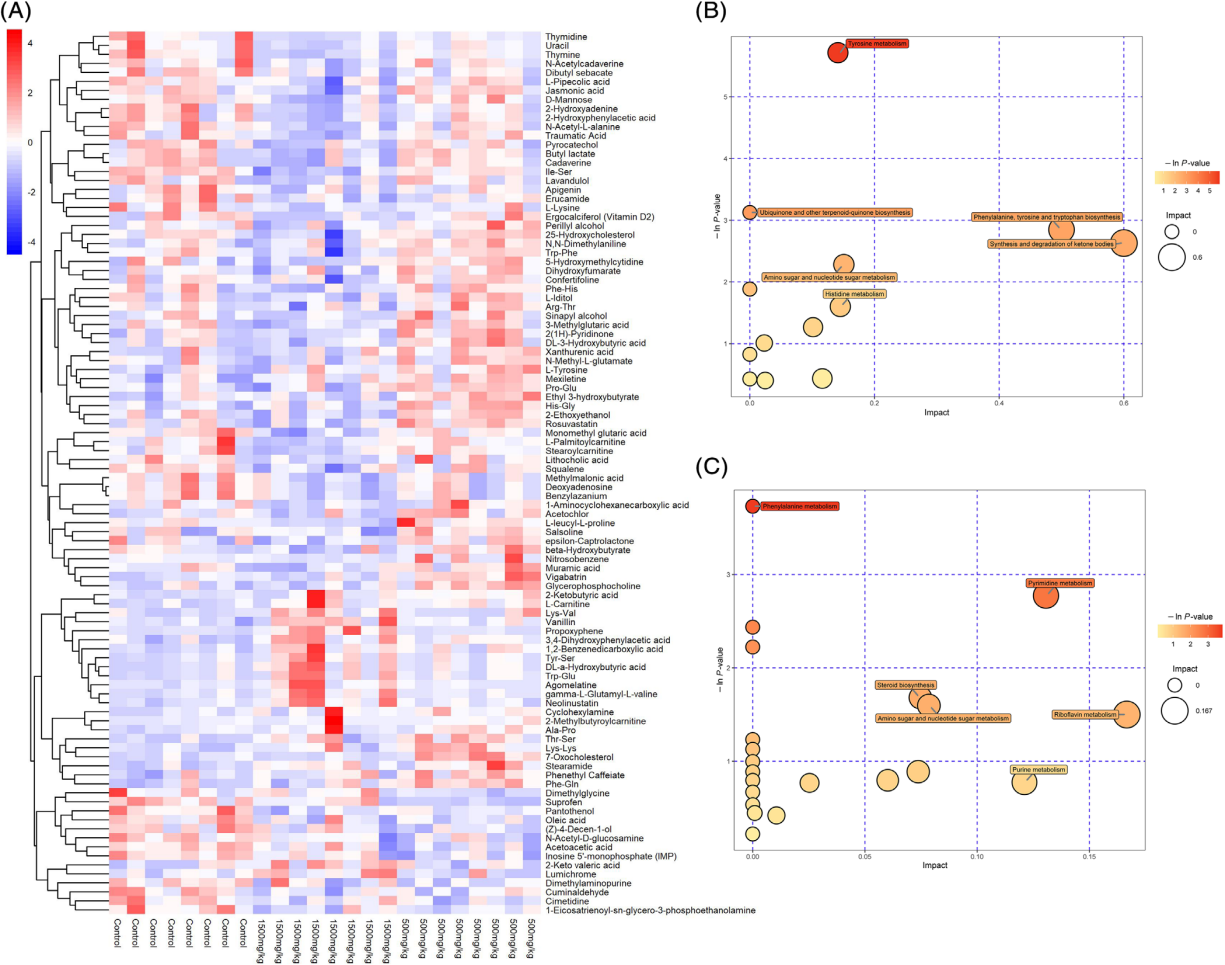
Differentially altered metabolites and their enriched signaling pathways after DEHP exposure. (A) Heatmap of the significantly altered metabolites in the control, 500 and 1500 mg/kg DEHP‐exposed groups. Individual samples (horizontal axis) and metabolites (vertical axis) are separated using hierarchical clustering. The color scale is noted in the upper left corner. Red and blue colors represent increased and decreased metabolites, respectively, relative to the median metabolite level. (B‐C) Bubble diagrams of the metabolic pathway topology analysis. The −ln(p) values from the pathway enrichment analysis are indicated on the horizontal axis, and impact values are indicated on the vertical axis. The colors and sizes of the shapes represent the effects of the 500 mg/kg (B) or 1500 mg/kg (C) DEHP treatments on metabolism relative to the control treatments, and the larger red shapes indicate a greater effect on the pathway [Color figure can be viewed at wileyonlinelibrary.com]

A heat map of three groups was constructed to visualize the results of the major abundant metabolites and summarize the distributions of the most significantly differentially altered metabolites distinguishing the three groups (Figure 4A). In addition, the differentially altered metabolites are listed in Table 1, and the levels of those metabolites were significantly different in the 500 and 1500 mg/kg DEHP groups compared with the control groups. These metabolites were products of carbohydrate metabolism, protein digestion and absorption, and fatty acid metabolism. The enrichment of the differentially abundant metabolites between the control and 1500 mg/kg DEHP‐exposed groups was determined by analyzing Kyoto Encyclopedia of Genes and Genomes (KEGG) pathways, and the differentially abundant metabolites were related to some metabolic pathways. In the comparison of the control and 500 mg/kg DEHP‐exposed groups, tyrosine metabolism, ubiquinone and other terpenoid‐quinone biosynthesis, amino sugar and nucleotide sugar metabolism, histidine metabolism, phenylalanine, tyrosine and tryptophan biosynthesis, and synthesis and degradation of ketone bodies were the affected pathways (Figure 4(B)). In the comparison of the control and 1500 mg/kg DEHP‐exposed groups, phenylalanine metabolism, steroid biosynthesis, amino sugar and nucleotide sugar metabolism, purine metabolism, pyrimidine metabolism, and riboflavin metabolism were affected (Figure 4(C)). In the comparison of the 500 and 1500 mg/kg DEHP‐exposed groups, pyrimidine metabolism, riboflavin metabolism, pyruvate metabolism, porphyrin and chlorophyll metabolism, and amino acid (valine, leucine and isoleucine) biosynthesis were affected (Figure S5). In addition, the differentially altered metabolites enriched in these signaling pathways are listed in Table S1.

**TABLE 1:** The differential metabolites in groups of 500 and 1500 mg/kg DEHP compared with the control

Metabolite of MS2	Control	500 mg/kg	1500 mg/kg
Dexpanthenol	0.081 ± 0.028	0.049 ± 0.010^a^	0.034 ± 0.015^a^
Theobromine	0.137 ± 0.072	0.325 ± 0.044^a^	0.285 ± 0.088^a^
4‐Pyridoxic acid	18.077 ± 3.112	23.352 ± 2.851^a^	41.810 ± 19.651^a^
Phthalic acid Mono‐2‐ethylhexyl Ester	190.677 ± 188.904	597.635 ± 386.909^a^	1049.460 ± 583.644^b^
Confertifoline	3.358 ± 2.581	7.598 ± 4.502^a^	11.806 ± 5.045^b^
4‐Hydroxybenzaldehyde	6.268 ± 1.188	7.784 ± 0.709^a^	13.881 ± 6.576^b^
Taurochenodeoxycholate	2.143 ± 1.176	1.394 ± 0.238^a^	1.048 ± 0.468
Hydroxyhydroquinone	0.516 ± 0.311	1.062 ± 0.577^a^	1.657 ± 0.762^b^
2‐Amino‐3‐methoxy‐benzoic acid	0.330 ± 0.106	0.523 ± 0.166^b^	0.221 ± 0.135^b^
5’‐Deoxyadenosine	1.748 ± 1.494	0.538 ± 0.349^b^	0.746 ± 0.725^b^
Acetylcarnitine	0.319 ± 0.080	0.500 ± 0.241^b^	1.061 ± .735^b^
Caprylic acid	2.066 ± 0.404	3.592 ± 1.447^b^	5.248 ± 3.399^b^
Suberic acid	0.152 ± 0.029	0.358 ± 0.205^b^	3.369 ± 3.828^b^
ketoisocaproic acid	40.847 ± 10.208	24.880 ± 10.699^b^	22.902 ± 16.736^a^
Propionylglycine	0.182 ± 0.154	0.282 ± 0.126^b^	0.524 ± 0.547^b^
2‐Ethyl‐2‐Hydroxybutyric acid	0.109 ± 0.039	0.143 ± 0.032^b^	0.291 ± 0.191^b^
4‐Methoxyphenylacetic acid	1.505 ± 0.844	3.092 ± 1.913^b^	0.882 ± 0.745^b^
2‐(4‐Hydroxyphenyl)ethanol	0.331 ± 0.081	0.438 ± 0.113^b^	0.428 ± 0.102^b^
2‐Methylbenzoic acid	1.373 ± 0.857	0.849 ± 0.307^b^	0.935 ± 0.578^b^
Creatinine	0.036 ± 0.010	0.146 ± 0.186^b^	0.097 ± 0.081^b^
Nitrendipine	0.029 ± 0.012	0.056 ± 0.027^b^	0.049 ± 0.024^b^
Glucosamine	0.140 ± 0.022	0.165 ± 0.021^b^	0.103 ± 0.039^b^

^a^ Denotes *p* < .01 between DEHP exposed and control, and,

^b^ Denotes *p* < .05 between DEHP exposed and control.

### 3.4 Correlation between the Fecal Microbiota and Metabolites

**FIGURE 5:**
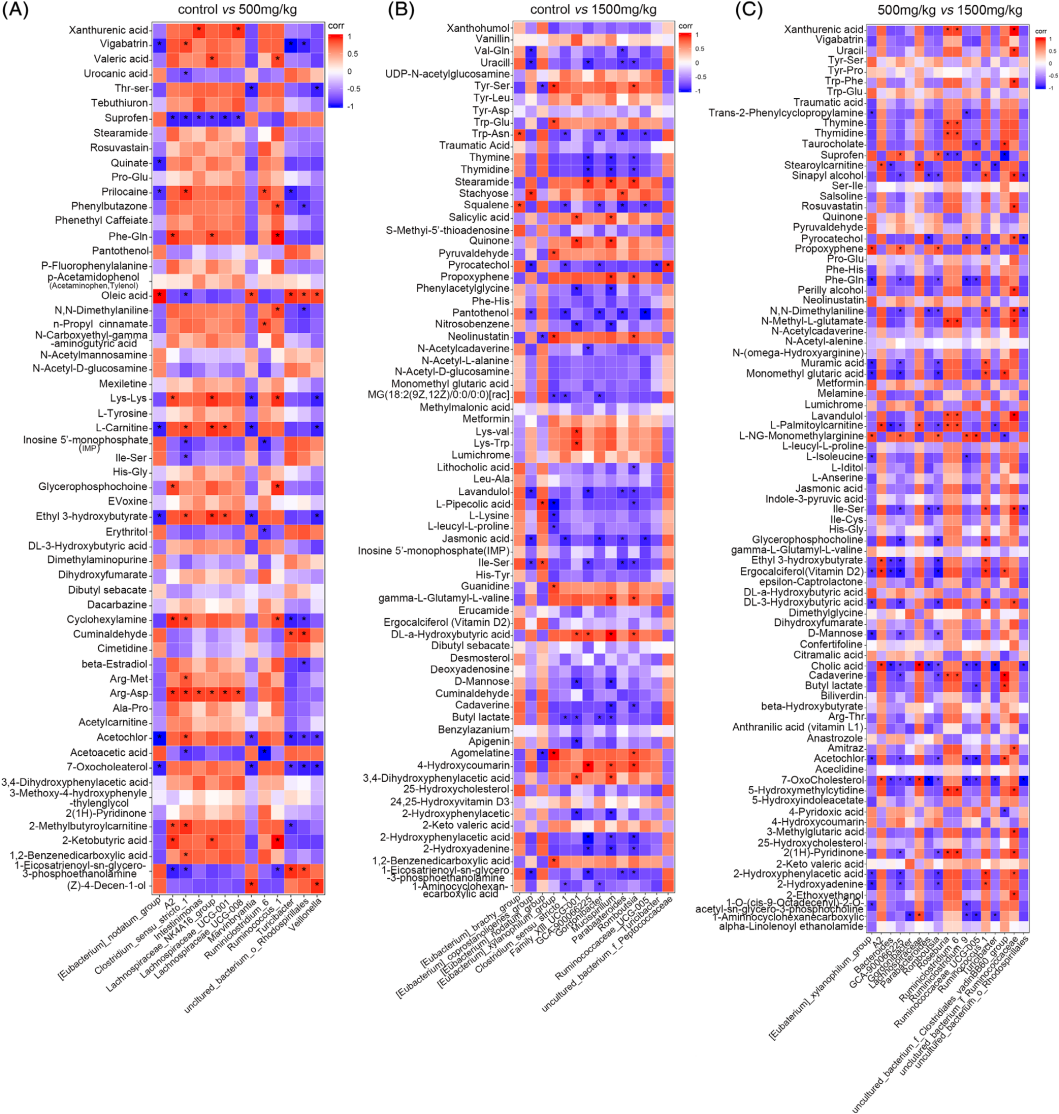
Correlations between the fecal microbiota and metabolites after DEHP exposure in pairwise comparison. (A) Control group vs. the 500 mg/kg DEHP group, (B) control group vs. the 1500 mg/kg DEHP group, and (C) 500 mg/kg DEHP group vs. 1500 mg/kg DEHP group. Spearman's rank correlation coefficients and *p* values for the correlations of fecal bacteria and their metabolites were calculated. Red represents a positive correlation and blue represents a negative correlation. *p < .05 [Color figure can be viewed at wileyonlinelibrary.com]

Spearman's correlation analysis was conducted in this study to further explore the functional correlations between the composition of the gut microbiota and the altered fecal metabolites. In the resulting heatmap, the analysis revealed strong correlations between several specific gut bacteria and typical metabolites. In the comparison of the control and 500 mg/kg DEHP‐exposed groups, ketobutyric acid and Phe‐Gln were positively correlated with *Ruminococcus*_1, acetoacetic acid negatively correlated with *Ruminiclostridium*_6, vigabatrin negatively correlated with *Turicibacter*, acetochlor negatively correlated with the [*Eubacterium*]_*nodatum*_group, and oleic acid positively correlated with the [*Eubacterium*]_*nodatum*_group (Figure 5(A)). In the comparison of the control and 1500 mg/kg DEHP‐exposed groups, GCA‐900066225 negatively correlated with 2‐hydroxyphenylacetic acid but positively correlated with 4‐hydroxycoumarin, DL‐α‐hydroxybutyric acid positively correlated with *Mucispirillum*, pantothenol negatively correlated with *Ruminococcaceae*_UCG‐005, and [*Eubacterium*]_*xylanophilum*_group negatively correlated with pipecolic acid but positively correlated with agomelatine (Figure 5(B)). In the comparison of the 500 and 1500 mg/kg DEHP groups, 7‐oxocholesterol displayed positive correlations with *Parabacteroides* and uncultured_bacterium_o_*Rhodospirillales*, lavandulol and indole‐3‐pyruvic acid displayed positive correlations with uncultured_bacterium_f_*Ruminococcaceae*, cholic acid negatively correlated with *Turicibacter* but positively correlated with the *Lachnospiraceae*_NK4A136_group, and the uncultured_bacterium_f_*Clostridiales*_vadinBB60_group positively correlated with cadaverine but negatively correlated with suprofen (Figure 5(C)). Based on these results, the changes in the gut bacterial compositions obviously and specifically altered the levels of bacterial metabolites in feces after DEHP exposure.

### 3.5 Oxidative stress in the ovary and systemic inflammation

**FIGURE 6:**
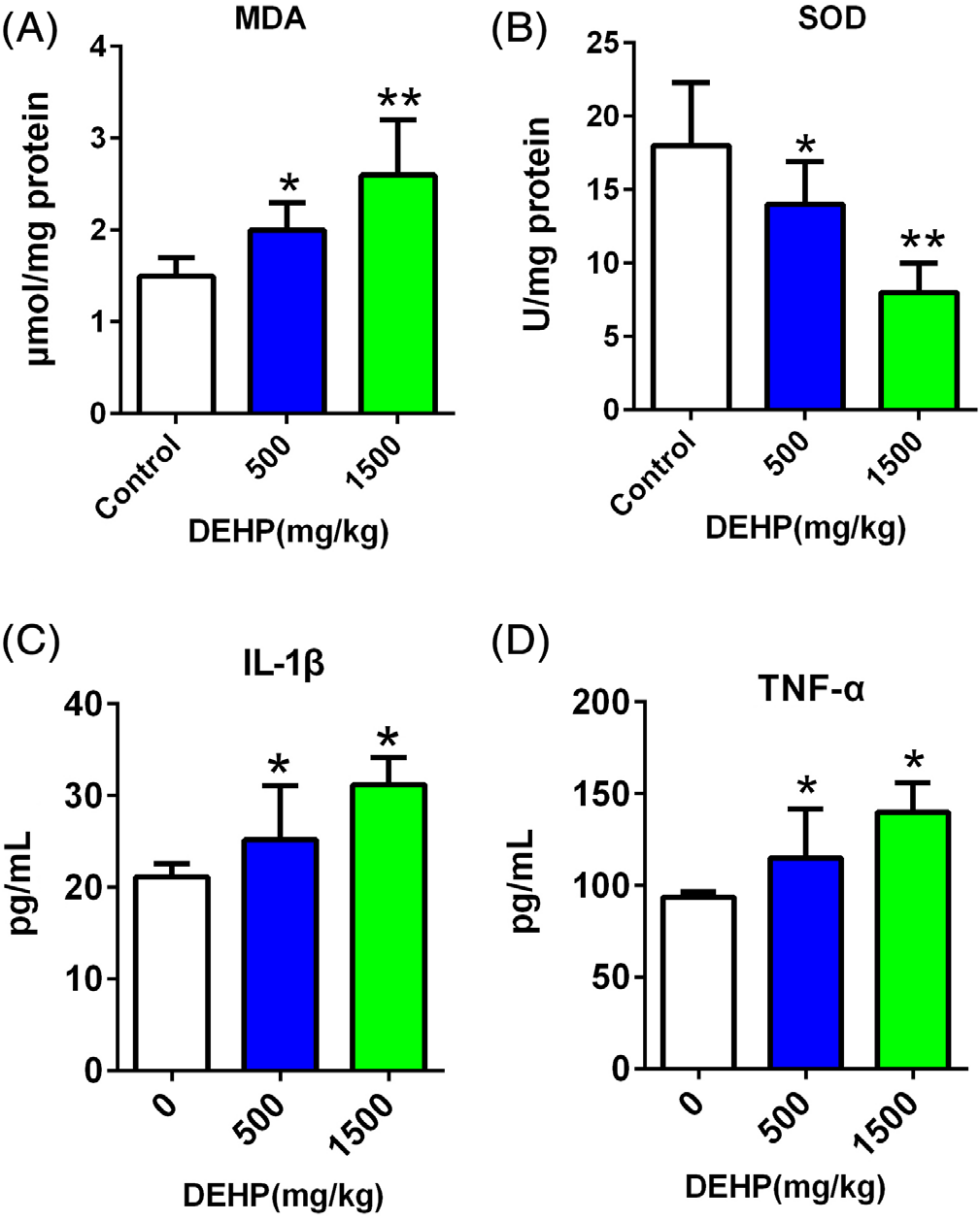
Oxidative stress in the ovary and systemic inflammation after DEHP exposure. (A) MDA levels increased in the ovary after DEHP exposure. (B) SOD levels in the ovary decreased after DEHP exposure. (C) Plasma IL‐1β levels increased after DEHP exposure. (D) Plasma TNF‐α levels increased after DEHP exposure. *p < .05 and **p < .01 [Color figure can be viewed at wileyonlinelibrary.com]

We detected the indicators of oxidative stress MDA and SOD in ovarian tissues and proinflammatory factors in the blood of DEHP‐exposed mice to elucidate the mechanism by which the DEHP‐induced changes in the fecal microbiota and metabolites cause female reproductive toxicity. The MDA concentration was increased (Figure 6(A)) and the SOD concentration was decreased (Figure 6(B)) in the DEHP‐exposed groups compared with the control group. The analysis of systemic proinflammatory factors showed that IL‐1β and TNF‐α levels were increased in mice after DEHP exposure (Figure 6(C) and (D)).

## 4 DISCUSSION

DEHP is one of the most environmentally abundant endocrine‐disrupting chemicals that cause reproductive abnormalities in humans, and DEHP can leach out of plastic beverage or food containers and readily enter the blood following oral ingestion. DEHP disrupts normal reproductive and ovarian function,^22^ alters follicular development during weaning and maturity,^38^ impairs the steroidogenesis of ovarian follicular cells,^39^ and induces premature ovarian failure.^40^ In addition, prenatal exposure to DEHP exerts multigenerational and transgenerational effects on female reproduction.^17^ In the present study, DEHP exposure altered the estrous cycle and estrogen level, accelerated primordial follicle recruitment and induced follicular atresia. In terms of toxicological mechanisms, DEHP induces DNA damage and apoptosis of the ovarian somatic cells, and alters the ovarian oxidative stress status.^41^ DEHP interferes with PI3K signaling and induces an acceleration of primordial follicle development; it also affects oocyte maturation and the embryogenesis process.^22^, ^42^ DEHP downregulates the expression of the germ cell markers *Stra8*, *Dazl*, and *Nobox*, delays the fetal oogenesis processes, inhibits the expression of the *Gdf9* and *Atm* genes, and leads to abnormal follicular growth and cell division.^43^ Additionally, DEHP induces oxidative stress by promoting ROS generation and inhibits steroid synthesis by modulating the expression of steroidogenic responsive genes in granulosa cells; it also activates the Bax/Bcl‐2 and the caspase‐3‐mediated mitochondrial apoptotic pathway to induce apoptosis.^44^ Thus, DEHP induces oxidative stress in ovary and alters ovarian function to induce female reproductive toxicity. However, the specific mechanisms underlying the toxic effects of DEHP are an area that is largely unexplored.

Based on accumulating evidence, the gut microbiota plays an important role in maintaining animal health, and an imbalance in the intestinal flora is known to be associated with different metabolic diseases. The gut microbiome modulates the estrogen level in the host by secreting β‐glucuronidase, an enzyme that suppresses the binding of estrogen to its receptors and its subsequent physiological downstream effects.^29^, ^45^ The composition of the gut microbiota was recently shown to play an important role in sexual dimorphism or sex‐specific differences mediated by estrogen, and estradiol and estrogen‐like compounds induce changes in the gut microbiome to improve the sexual dimorphism of subjects with metabolic syndrome.^46^ Females are more resistant to gut injury compared to their male counterparts, and gut injury is decreased in male mice after androgen inhibition; therefore, the authors postulated that the gut epithelial barrier integrity was modified by estrogen.^47^ Baker et al. published a detailed review showing that the gut microbiome is the principal regulator of circulating estrogen levels and described the relationship between gut microbes and estrogen‐modulated diseases.^28^ According to these studies, homeostasis of the gut microbiome is closely related to normal estrogen levels; however, the causal relationship between these factors requires further study. Meanwhile, the role of estrogens in female reproductive system development and maintenance is well defined.^48^ In the present study, DEHP did not change the body weight that similar with a study of DEHP (750 mg/kg/d for 30 days) affected female reproduction, while DEHP (300 mg/kg/d for 4 weeks) could increase obesity‐induced damage to the male reproductive system in mice,^22^, ^49^ in addition, a study showed males and females respond differently to DEHP not only in age but also in non‐monotonic dose–response curve,^50^ it suggested that the effect of DEHP on body weight is related to gender and this need be further studied. DEHP affected estrogen levels and induced ovarian damage, consistent with other studies.^39^, ^51^ However, researchers had not clearly determined whether DEHP‐induced female reproductive toxicity mediates changes in the gut microbiome and its metabonomic mechanisms, and the effects of DEHP on the gut microbial and metabolomics profile and the relationships among these factors. In our study, we performed a microbiome/metabolome‐wide association study to reveal the effects of DEHP on the bacterial communities and fecal metabolomic profile in mice and mechanisms by which DEHP induced female reproductive toxicity by employing 16S rDNA gene sequencing and a nontargeted UHPLC‐QTOFMS analysis. The major findings from this study are listed below. (a) DEHP decreased the fecal microbiota diversity and modulated the composition of the microbiota in a manner that was detrimental to female reproductive health. (b) DEHP intake exerted a substantial effect on fecal metabolomics pathways associated with phenylalanine metabolism, steroid biosynthesis, amino sugar and nucleotide sugar metabolism, purine metabolism, pyrimidine metabolism, and riboflavin metabolism. (c) Some of the specifically altered specific gut microflora‐related metabolites were strongly associated with perturbed gut microbes.

The human body is reported to be exposed to DEHP at concentrations of up to 30 mg/kg/day, and occupational exposure levels of up to 600 mg/kg/d have been reported.^15^, ^18^ The doses to which the mice in present study were exposed were primarily selected based on the equivalent dose ratio calculated using the surface areas of humans and mice, and this exposure dose is consistent with human exposure in toxicological studies and might enable us to better assess the toxicological health effects. In the present study, the concentrations of DEHP to which mice were exposed were 500 and 1500 mg/kg. Although the a 3‐fold difference existed between the these two concentrations, the toxic effects, including estrogen levels, organ coefficient, oxidative stress and pro‐inflammatory factors, did not show a 3‐fold relationship, which may be related to the the non‐monotonic dose–response of DEHP.^50^ Despite the widespread exposure of the general population to DEHP, limited data are available describing the potential effects on the ovary or follicles in the female reproductive system in the general population mediated by perturbations in gut microbiome homeostasis. However, to the best of our knowledge, few studies have reported the changes in the gut microbiome following occupational exposure to DEHP. In addition, the changes in complicated human gut microbes are difficult to assess because various factors affect the microbiota, and a large number of samples must be repeatedly analyzed. Compared with humans, the use of a single diet and lower levels of differences in the gut microbiome between individual mice suggest that these animals are a better model to study the DEHP‐induced female reproductive toxicity mechanisms mediated by changes in the microbial composition. In the present study, after 30 days of oral DEHP exposure, the microbiota analysis showed significant differences in fecal microbial structures among the control, 500 mg/kg and 1500 mg/kg DEHP groups. DEHP significantly increased the abundances of *Firmicutes*, *Akkermansia*, *Turicibacter*, *Romboutsia* and *Verrucomicrobiales*, and decreased the abundances of *Bacteroidetes*, *Actinobacteria*, and *Epsilonbacteraeota* compared to the control group. An increase in the *Firmicutes/Bacteroidetes* (F/B) ratio in the gut of the mice exposed to DEHP may be responsible for the female reproductive toxicity, and the increase in the F/B ratio has been suggested as one of the hallmarks of reduced estrogen levels observed in senescent rats in a previous study.^52^ Data obtained from animal and preclinical studies showed that the F/B ratio is specifically increased in obese individuals.^53^, ^54^ In addition, obesity correlated with a reduction in estrogen levels in a previous study.^55^ A decrease in the abundance of beneficial bacteria and an increase in the abundance of pathogenic populations induce inflammation and are closely relate to the absence of estrogen.^28^, ^56^ A study showed that the F/B ratio and *Escherichia coli* abundance were higher in ovariectomized animals than normal females.^57^ Estrogen not only could increased abundance of *Akkermansia* and *Bifidobacterium* but also could inhibit the overgrowth of *Proteobacteria* and *Escherichia coli*.^58^ These studies similar with our studies indicated that the bidirectionality effects between the reproductive system and gut microbiota, however, the chronological relationship between estrogen and gut microbiota caused by DEHP still needs to be further proved.

In the present study, the abundances of *Verrucomicrobia* and *Akkermansia* were significantly increased in 1500 mg/kg DEHP‐exposed mice. *Akkermansia* is a member of the phylum *Verrucomicrobia* that is involved in the process of lipid metabolism. In previous studies, the abundance of *Verrucomicrobia* was significantly increased in monkeys with alcoholic fatty liver and nonobese mice fed a high‐fat diet.^59^, ^60^ In contrast, the abundance of *Verrucomicrobia* was significantly decreased in obese mice and patients with non‐alcoholic fatty liver cirrhosis.^61^, ^62^ According to Wang et al., astaxanthin increases the abundance of *Verrucomicrobia*, particularly *Akkermansia*, by regulating lipid metabolism and the gut microbiota to prevent obesity caused by a high‐fat diet.^63^ Derrien et al. reviewed the role of *Akkermansia* in regulating host functions and suggested that this genus may have potential anti‐inflammatory properties.^64^ Kaliannan et al. considered that estrogen obviously increased the abundance of lipopolysaccharide‐suppressing bacteria such as *Akkermansia* and significantly suppressed the development of endotoxemia and inflammation.^46^ DEHP increases weight gain and alters lipid metabolism and estrogen levels.^65^ *Turicibacter* and *Romboutsia* play important roles in lipid metabolism.^66^ Based on these studies, DEHP may alter estrogen levels by modulating lipid metabolism and the intestinal bacteria; however, the detailed mechanisms underlying this process require further study. *Turicibacter* belong to an extensively branched class of the phylum *Firmicutes* and have a close relationship with obesity and inflammation.^67^, ^68^ In the study by Caslin et al., a diet containing alcohol affected the key components of the microbiota responsible for immune regulation, including *Turicibacter* and *Akkermansia*.^69^ The abundance of intestinal bacteria such as *Epsilonbacteraeota*, *Bacteroidetes*, and *Actinobacteria* was significantly reduced, but the abundance of *Firmicutes* was significantly increased in a recent study after inflammation and lung injury were treated with a Chinese medicine.^70^ In the present study, the abundances of *Helicobacter*, *Campylobacter*, *Lachnospiraceae* and *Erysipelotrichia* were increased in DEHP‐exposed groups, according to the LEfSe analysis and LDA score. *Helicobacter* is a genus of gram‐negative bacteria and its major cell wall component lipopolysaccharide (LPS) induces systemic effects and even modifies lipid profiles, stimulates vascular inflammation, and exacerbates atherogenesis.^71^ Additionally, the most widely known species *Helicobacter pylori* is strongly associated with gastric cancer in Asia.^72^ *Campylobacter* is a gram‐negative bacteria that induces diarrhea.^73^ Members of the family *Lachnospiraceae*, such as *Ruminococcus gnavus*, express superantigen and activate the IgA response, which play a critical role in intestinal homeostasis.^74^ In addition, *Ruminococcaceae* and *Lachnospiraceae* participate in protecting against *Clostridium difficile* infection.^75^ The loss of protective gut commensal strains of the family *Lachnospiraceae* and an increase in the abundance of colitogenic strains of the family *Erysipelotrichaceae* in *Nlrp12*‐deficient mice causes colonic inflammation.^76^ Here, alterations in the bacterial composition of the gut represent one possible explanation for DEHP‐induced female reproductive toxicity and systemic inflammation due to increased LPS levels; however, in‐depth research must be conducted to verify this hypothesis. Gut bacterial dysbiosis may alter the bacterial metabolites and weaken the tight junctions, allowing paracellular translocation of lymphocytes and endotoxins into the ovary via the general circulation. These endotoxins may increase the levels of the proinflammatory cytokines IL‐1β, IL‐18, and tumor necrosis factor‐α by activating Toll‐like receptors (TLRs). These cytokines may subsequently reach the ovary to exert direct effects on ovarian follicular cells, accompanied by increased inflammation and oxidative stress. In addition, IL‐22 plays a critical role in bile acid‐mediated regulation of the gut microbiota in individuals with polycystic ovary syndrome.^77^, ^78^ Thus, the activation of the inflammatory response and oxidative stress caused by intestinal bacteria imbalance may be the important factors of damage to the female reproductive system.

The importance of short chain fatty acids (SCFAs) such as propionate and butyrate has been confirmed in human studies. In the present study, the levels of fecal metabolites, such as SCFAs (acetic acid, propionic acid and butyric acid), amino acids and simple sugars, were significantly altered and exhibited strong correlations with gut microbiota dysbiosis after DEHP exposure, as well as the altered microbes, including *Ruminiclostridium*, *Clostridiales*, *Turicibacter*, Lachnospiraceae, Verrucomicrobia, *Ruminococcus*, *Parabacteroides*, *Roseburia*, *Clostridium*, *Intestinimonas* and *Eubacterium*, etc. A change in the abundance of *Ruminiclostridium* is closely related to the production of SCFAs and inhibition of the endotoxin LPS, and *Ruminiclostridium* is one of the dominant genera responsible for volatile fatty acid production.^33^, ^79^ The SCFA‐producing bacteria of the gut microbiota, such as *Bacteroides*, *Lactobacillus*, and *Lachnospiraceae*, show negative correlations with fecal LPS concentrations.^80^ An increase in the abundance of SCFA‐producing bacteria might reduce inflammation.^43^ The concentration of butyrate decreased after propionate was infused into the caecum of pigs, the abundance of *Bacteroidetes* increased, the abundance of *Firmicutes* decreased, tyramine levels increased, cadaverine levels decrease, and the expression of proinflammatory factors was upregulated, suggesting that butyrate and propionate exert opposite effects on regulating inflammation by modulating the gut bacterial ecology.^81^ Additionally, although some studies suggest that propionic acid may be the main cause of pathopoiesia, other studies indicate that the production of propionic acid in the intestinal tract maintains the homeostasis of the gut microbiota.^82^, ^83^, ^84^, ^85^ Therefore, the role of propionic acid is closely related to the proportion of other organic acids in the intestinal environment, which requires further study. The relative abundances of *Roseburia*, *Ruminococcus* and *Clostridium* were directly correlated with total SCFA, butyrate, and propionate levels, and *Roseburia* and *Intestinimonas* are important producers of propionate and butyrate, respectively^86^, ^87^ In addition, a change in the F/B ratio is directly related to SCFA metabolism.^88^ Butyrate plays an important role in improving colonic defense barriers by increasing the expression of tight junction proteins, and the DEHP‐induced increase in the levels of inflammatory factors may be partially due to the reduction in butyrate and subsequent increase in colonocyte permeability.^89^ A study showed the changes of fecal microbiota composition and metabolites are related to the plasma reproductive hormones during pregnant and lactating stages in Bama mini pigs model.^90^ The SCFA not only regulates the estradiol secretion but also participates in endogenous estrogen receptor‐alpha‐mediated signaling.^91^, ^92^ These result implied that the SCFAs potentially play an important role in DEHP‐induced female reproductive toxicology. Overall, the reduction in the SCFA levels after DEHP exposure may be due to gut bacterial dysbiosis and alterations in gut permeability. In a study reporting that perinatal bisphenol A exposure induces chronic inflammation in rabbit offspring, the authors found that females exhibited more severe inflammation than males due to the overexpression of estrogen receptor‐2.^93^ In addition, both bisphenol A and DEHP have estrogen interference effect in reproductive toxicity.

Many fecal metabolites generated by the gut microbiota, particularly benzene homologs and their derivatives, induce a systemic inflammatory response and oxidative stress.^94^ Phenylacetic acid is related to a more pro‐oxidant and immune‐stimulated status, which are both negatively associated with fecal propionate levels, whereas phenylpropionic acid is directly related to the fecal acetate level.^95^ Leucine, isoleucine and valine are essential amino acids termed branched‐chain amino acids due to the presence of an aliphatic side‐chain, and high concentrations of branched‐chain amino acids potentially exert deleterious effects and induce a pro‐inflammatory and oxidative stress status.^96^ We detected oxidative stress and systemic inflammation in ovarian tissues to clarify the mechanism of DEHP and involving the intestinal microbiota and fecal metabolites in female reproductive toxicity. As expected, oxidative stress and inflammation were increased after DEHP exposure. Thus, oxidative stress in the ovary and systemic inflammation may be the main factors contributing to female reproductive toxicity, which may be caused by changes in the intestinal flora and fecal metabolites following DEHP exposure. Therefore, the levels of the amino acids leucine, isoleucine and valine were significantly altered by DEHP exposure, according to feces metabolomic profile, and these amino acids may be involved in systemic inflammation and oxidative stress. Moreover, DEHP‐induced female reproductive toxicity and ovarian damage might be due to increased levels of oxidative stress and inflammation caused by metabolites from feces. Although the levels of many potential metabolic markers were increased after DEHP exposure at the doses used in this study, further studies are required to determine whether these metabolites are directly related to the inflammatory response and oxidative stress.

## 5 CONCLUSION

DEHP exposure induced reproductive toxicity and significantly altered the composition of gut microbes and fecal metabolites. Furthermore, one of the possible reasons is that DEHP‐induced female reproductive toxicity caused by gut bacterial dysbiosis and altered gut metabolite profiles, and potentially continuous DEHP exposure in women of child‐bearing ages might result in alterations in the gut bacterial composition and reductions in the levels of beneficial bacterial metabolites, such as SCFAs. Our studies also revealed the novel mechanism by which DEHP causes female reproductive toxicity through alterations in the fecal microbiota and metabonomics, and specific components of the fecal microbiota and metabolites might represent a microbial signature that correlates with DEHP exposure. Additionally, this study would also provide a basic reference for studies evaluating the reproductive toxicity of other environmental endocrine disruptors.

## CONFLICT OF INTEREST

The authors declare no potential conflict of interest.

## Supporting information


**Figure S1** Alpha diversity analysis of gut microbial species in mice exposed to 500 mg/kg and 1500 DEHP mg/kg. (A) The Chao1 index indicates the species number in each sample. (B) Rarefaction curves based on the observed species values. (C) Shannon indices were calculated to show that the data cover all species in the gut microbial community. (D) The rank abundance curve reflects the richness and evenness of the species in each sample.

**Figure S2** The taxonomic distributions of the fecal microbiota at the class (A), family (B), and genus (C) levels following exposure to DEHP.

**Figure S3** Taxonomic cladogram obtained from the LEfSe analysis. (A and C) The taxonomic cladograms of pairwise comparisons three groups, and the blue and orange dots are proportional to the degree of enrichment of certain taxa between two comparative groups (A: control group vs the 1500 mg/kg DEHP‐exposed group; C: 500 mg/kg DEHP‐exposed group vs 1500 mg/kg DEHP‐exposed group). (B and D) Taxa with LDA scores greater than 4 (B: control group vs the 1500 mg/kg DEHP‐exposed group; D: 500 mg/kg DEHP‐exposed group vs 1500 mg/kg DEHP‐exposed group). Taxa are represented as c (class), o (order), f (family), and g (genus).

**Figure S4** Permutation test of the OPLS‐DA model for the comparison of the control and 1500 mg/kg DEHP groups (A) and 500 mg/kg and 1500 mg/kg DEHP groups (C). Volcano plots of the control and 1500 mg/kg DEHP groups (B) and 500 mg/kg and 1500 mg/kg DEHP groups (D).

**Figure S5** Bubble diagrams of the metabolic pathway topology analysis of the 500 mg/kg and 1500 mg/kg DEHP‐exposed groups. The −ln(p) values from the pathway enrichment analysis are indicated on the horizontal axis, and impact values are indicated on the vertical axis. The colors and sizes of the shapes represent the effects of the 1500 mg/kg DEHP treatments on metabolism relative to the 500 mg/kg DEHP treatments, and the larger red shapes indicate a greater effect on the pathway.

**Table S1** The differentially metabolites enriched in KEGG pathways

## Data Availability

The data that support the findings of this study are available from the corresponding author upon reasonable request
