# Supplementary figures and images for: Di‐(2‐ethylhexyl) phthalate exposure induces female reproductive toxicity and alters the intestinal microbiota community structure and fecal metabolite profile in mice
Source: Environ Toxicol. 2021 Mar 4;36(6):1226–42. doi: 10.1002/tox.23121 (PMC8251547; doi:10.1002/tox.23121)

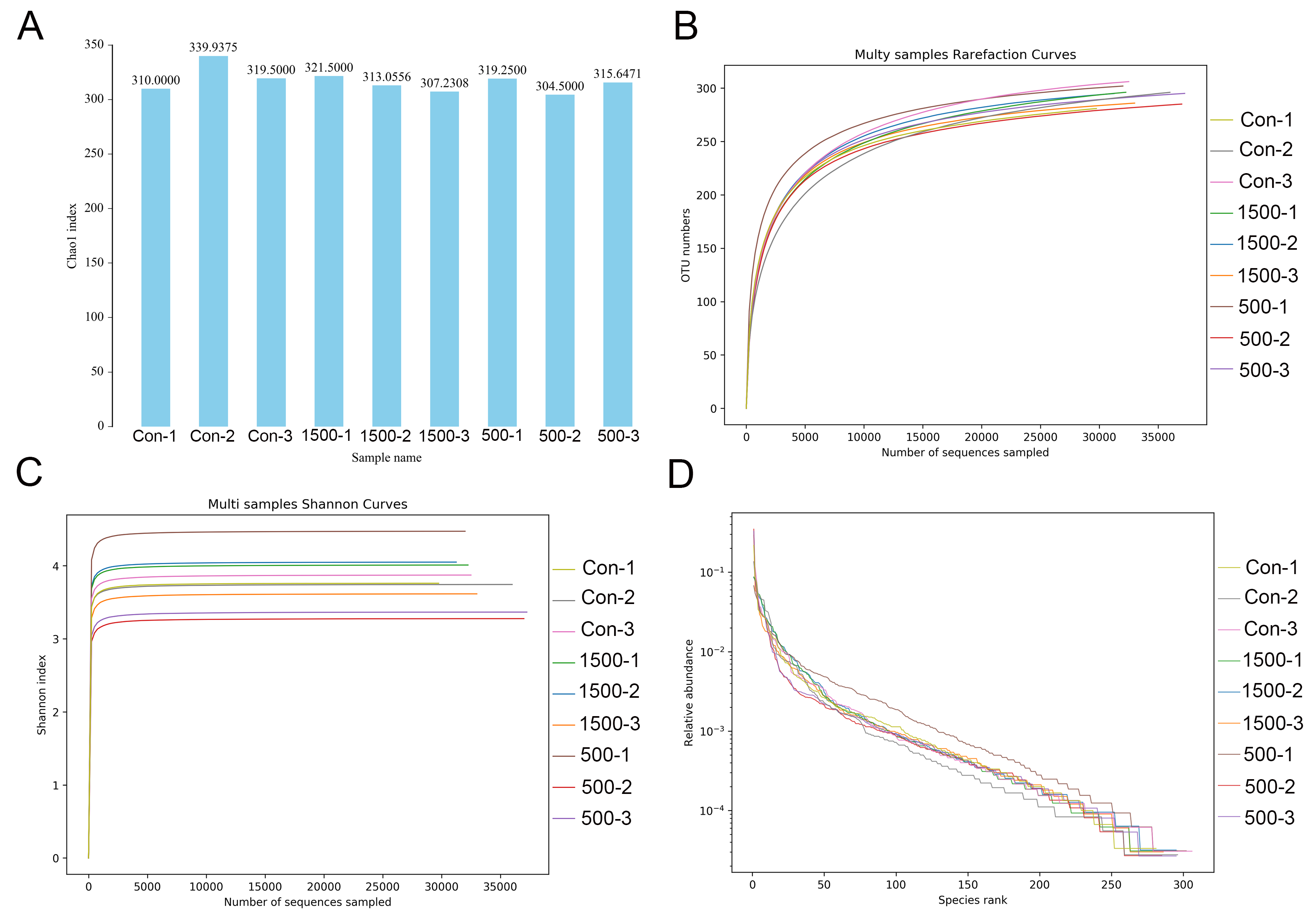

Supplement: Supplementary file 1 — Figure S1 Alpha diversity analysis of gut microbial species in mice exposed to 500 mg/kg and 1500 DEHP mg/kg. (A) The Chao1 index indicates the species number in each sample. (B) Rarefaction curves based on the observed species values. (C) Shannon indices were calculated to show that the data cover all species in the gut microbial community. (D) The rank abundance curve reflects the richness and evenness of the species in each sample. [file TOX-36-1226-s005.tif]

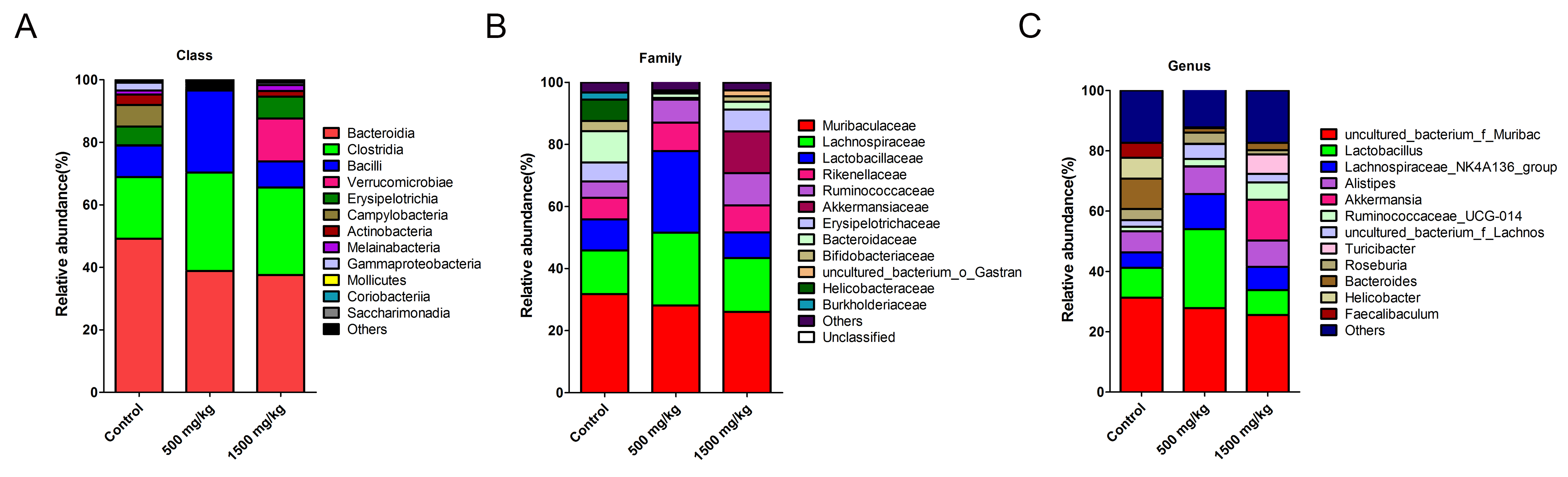

Supplement: Supplementary file 2 — Figure S2 The taxonomic distributions of the fecal microbiota at the class (A), family (B), and genus (C) levels following exposure to DEHP. [file TOX-36-1226-s004.tif]

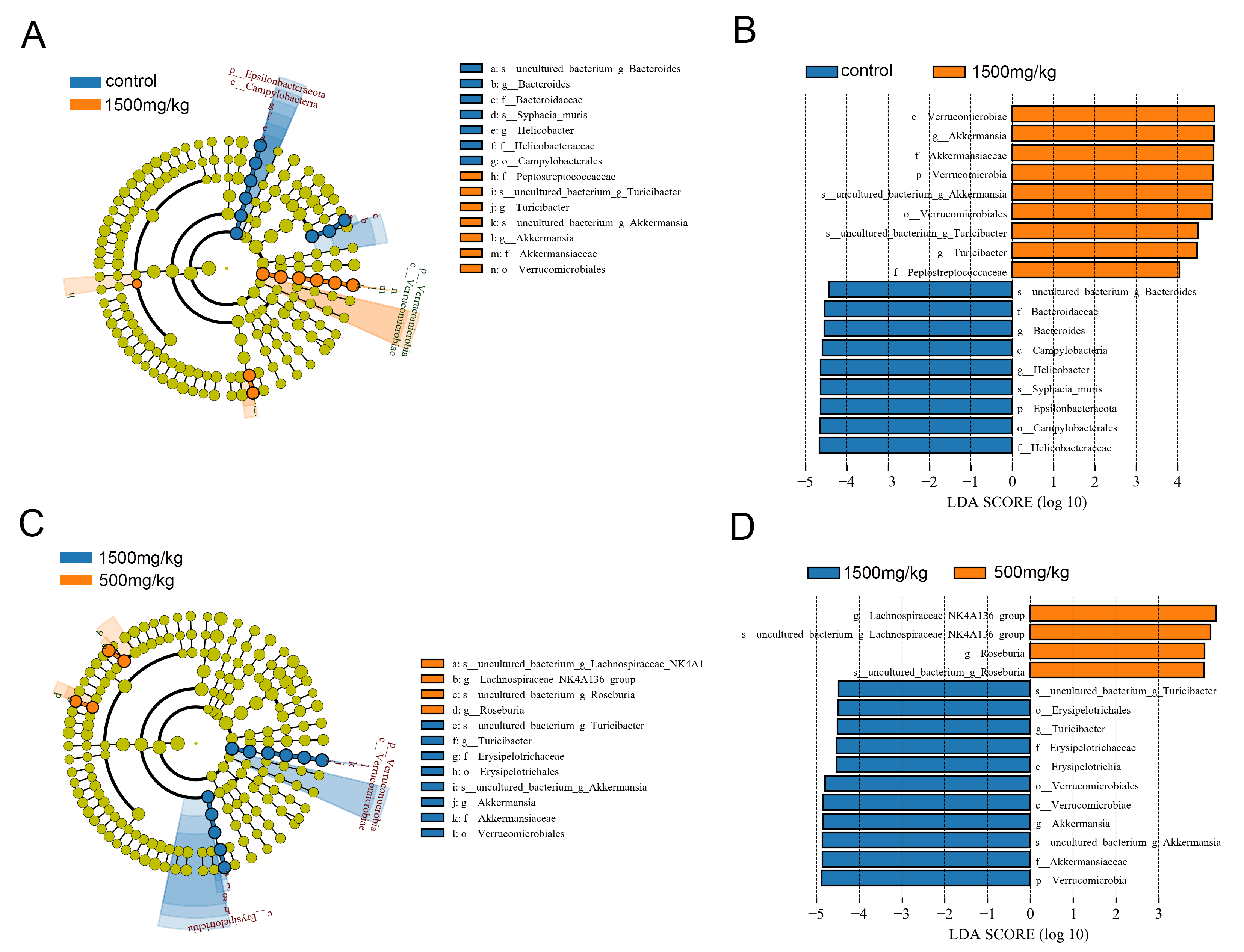

Supplement: Supplementary file 3 — Figure S3 Taxonomic cladogram obtained from the LEfSe analysis. (A and C) The taxonomic cladograms of pairwise comparisons three groups, and the blue and orange dots are proportional to the degree of enrichment of certain taxa between two comparative groups (A: control group vs the 1500 mg/kg DEHP‐exposed group; C: 500 mg/kg DEHP‐exposed group vs 1500 mg/kg DEHP‐exposed group). (B and D) Taxa with LDA scores greater than 4 (B: control group vs the 1500 mg/kg DEHP‐exposed group; D: 500 mg/kg DEHP‐exposed group vs 1500 mg/kg DEHP‐exposed group). Taxa are represented as c (class), o (order), f (family), and g (genus). [file TOX-36-1226-s002.tif]

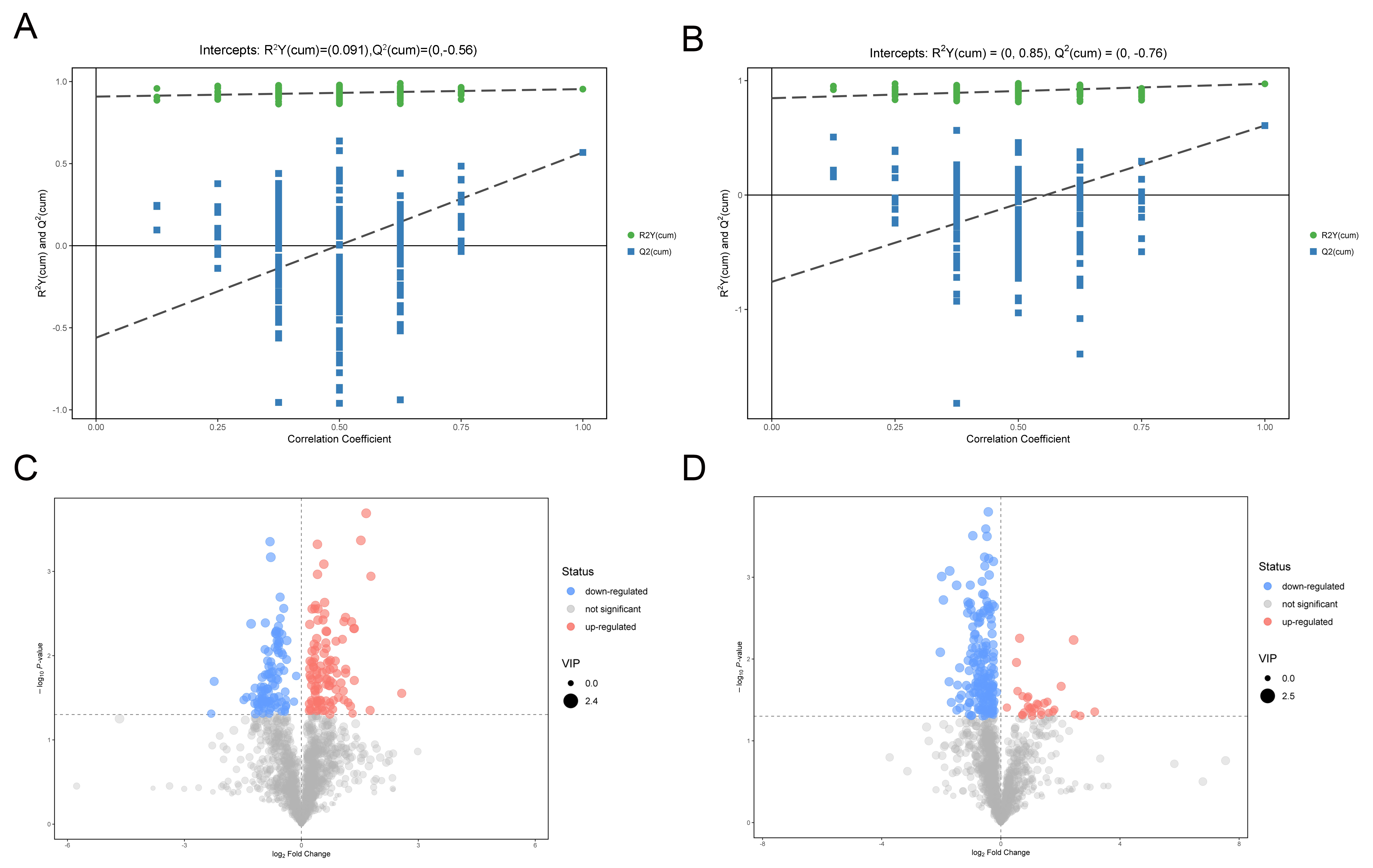

Supplement: Supplementary file 4 — Figure S4 Permutation test of the OPLS‐DA model for the comparison of the control and 1500 mg/kg DEHP groups (A) and 500 mg/kg and 1500 mg/kg DEHP groups (C). Volcano plots of the control and 1500 mg/kg DEHP groups (B) and 500 mg/kg and 1500 mg/kg DEHP groups (D). [file TOX-36-1226-s001.tif]

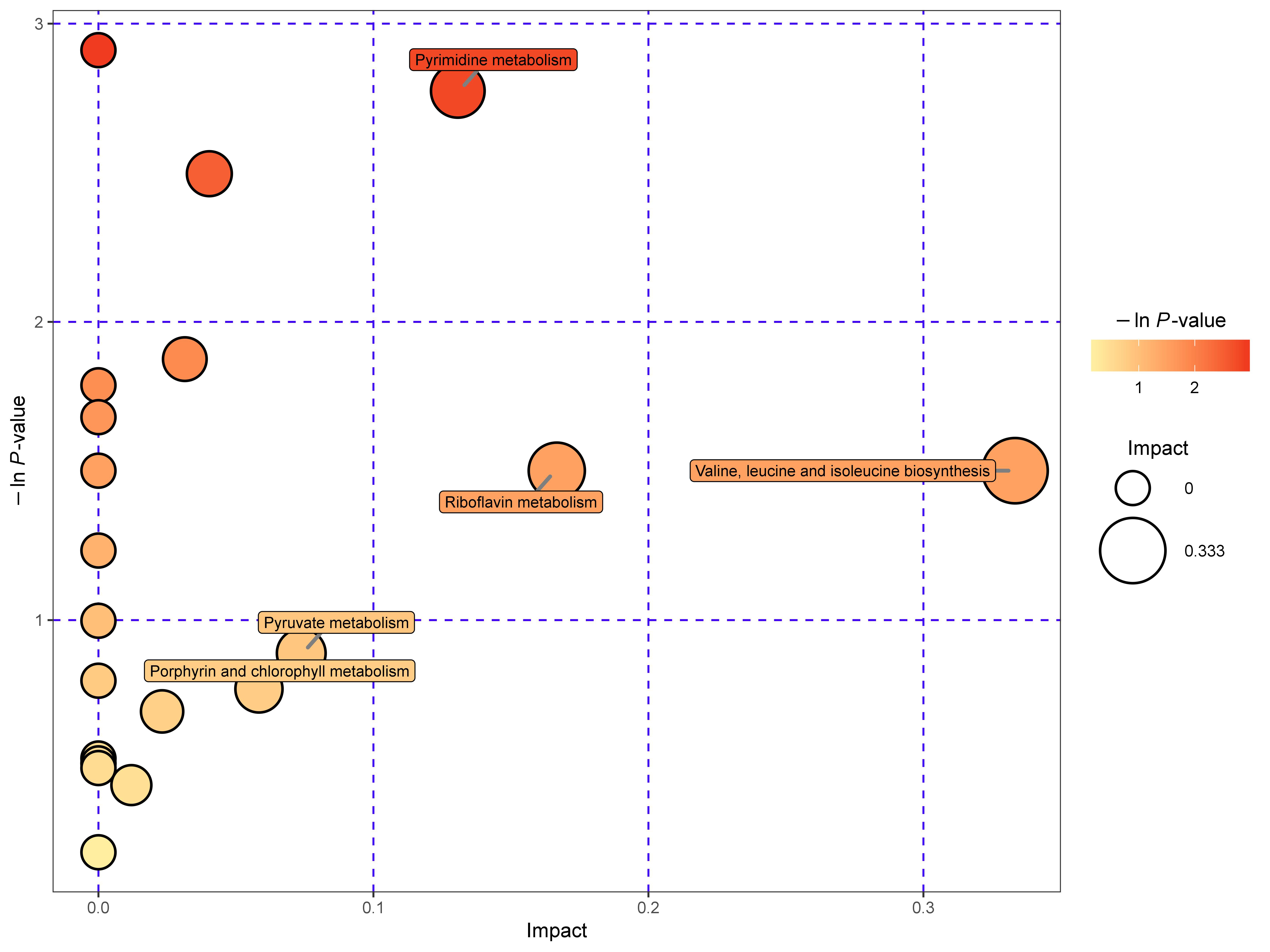

Supplement: Supplementary file 5 — Figure S5 Bubble diagrams of the metabolic pathway topology analysis of the 500 mg/kg and 1500 mg/kg DEHP‐exposed groups. The −ln(p) values from the pathway enrichment analysis are indicated on the horizontal axis, and impact values are indicated on the vertical axis. The colors and sizes of the shapes represent the effects of the 1500 mg/kg DEHP treatments on metabolism relative to the 500 mg/kg DEHP treatments, and the larger red shapes indicate a greater effect on the pathway. [file TOX-36-1226-s003.tif]
