# Supplementary material for: Di‐(2‐ethylhexyl) phthalate exposure induces female reproductive toxicity and alters the intestinal microbiota community structure and fecal metabolite profile in mice
Source: Environ Toxicol. 2021 Mar 4;36(6):1226–42. doi: 10.1002/tox.23121 (PMC8251547; doi:10.1002/tox.23121)
Supplement: Supplementary file 6 — Table S1 The differentially metabolites enriched in KEGG pathways [file TOX-36-1226-s006.doc]

| Table S1. The differentially metabolites enriched in KEGG pathways | |
| --- | --- |
| **Pathways** | **Total metabolites** |
| Phenylalanine metabolism | Phenylacetaldehyde; Phenylacetyl-CoA; L-Phenylalanine; Phenylethylamine; Phenylpyruvic acid; Phenylacetic acid; Phenylacetylglycine; Ortho-Hydroxyphenylacetic acid; Enol-phenylpyruvate; 2-Phenylacetamide; L-Tyrosine |
| Ubiquinone and other terpenoid-quinone biosynthesis | 4-Hydroxyphenylpyruvic acid; L-Tyrosine; Homogentisic acid |
| Pyrimidine metabolism | Thioredoxin; Uridine 5'-diphosphate; L-Glutamine; Carbamoylphosphate; 4,5-Dihydroorotic; Orotidylic acid; RNA; Uridine triphosphate; Uridine 5'-monophosphate; Uridine; Dihydrouracil; Ureidopropionic acid; Cytidine triphosphate; CDP; Cytidine monophosphate; Cytidine; Thioredoxin disulfide; dCDP; dCTP; dCMP; Deoxycytidine; Deoxyuridine triphosphate; dUDP; dUMP; Deoxyuridine; Thymidine 5'-triphosphate; dTDP; 5-Thymidylic acid; Thymidine; Dihydrothymine; Ureidoisobutyric acid; P1,P4-Bis(5'-uridyl) tetraphosphate; Ureidosuccinic acid; Phosphoribosyl pyrophosphate; Orotic acid; Uracil; Beta-Alanine; DNA; Deoxyribose 1-phosphate; Thymine; 3-Aminoisobutanoic acid |
| Lysine biosynthesis | Saccharopine; Aminoadipic acid; L-Lysine; Oxoadipic acid |
| Phenylalanine, tyrosine and tryptophan biosynthesis | Phenylpyruvic acid; L-Phenylalanine; L-Tyrosine; 4-Hydroxyphenylpyruvic acid |
| Tyrosine metabolism | L-Dopachrome; Normetanephrine; 3-Methoxy-4-hydroxyphenylglycolaldehyde; Norepinephrine; Epinephrine; 3,4-Dihydroxymandelate; 3,4-Dihydroxymandelaldehyde; 3,4-Dihydroxyphenylglycol; Metanephrine; Dopamine; 3,4-Dihydroxyphenylacetaldehyde; 3,4-Dihydroxybenzeneacetic acid; Homovanillin; 3-Methoxytyramine; L-Dopa; 3,5-Diiodo-L-tyrosine; Iodotyrosine; L-Tyrosine; 4-Fumarylacetoacetic acid; Maleylacetoacetic acid; Homogentisic acid; 4-Hydroxyphenylpyruvic acid; 4-Hydroxyphenylacetyl-CoA; 4-Hydroxyphenylacetaldehyde; Tyramine; 5,6-Dihydroxyindole; Gentisate aldehyde; N-Methyltyramine; 5,6-Dihydroxyindole-2-carboxylic acid; Vanillylmandelic acid; Vanylglycol; Homovanillic acid; Liothyronine; Fumaric acid; Acetoacetic acid; 2-Hydroxy-3-(4-hydroxyphenyl)propenoic acid; Hydroxyphenylacetylglycine; p-Hydroxyphenylacetic acid; Indole-5,6-quinone; Dopaquinone; Thyroxine; Gentisic acid; Hordenine; 5,6-Indolequinone-2-carboxylic acid |
| Biotin metabolism | Biotin; Biotinyl-5'-AMP; Biocytin; Holo-[carboxylase]; L-Lysine |
| Synthesis and degradation of ketone bodies | Acetoacetyl-CoA; Acetyl-CoA; 3-Hydroxy-3-methylglutaryl-CoA; (R)-3-Hydroxybutyric acid; Acetoacetic acid |
| Valine, leucine and isoleucine degradation | Enzyme N6-(lipoyl)lysine; 2-Methyl-1-hydroxybutyl-ThPP; Enzyme N6-(dihydrolipoyl)lysine; 2-Methyl-1-hydroxypropyl-ThPP; 3-Methyl-1-hydroxybutyl-ThPP; Acetyl-CoA; Acetoacetyl-CoA; Acetoacetic acid; 3-Hydroxy-3-methylglutaryl-CoA; 3-Methylcrotonyl-CoA; 3-Hydroxyisovaleryl-CoA; Isovaleryl-CoA; 3-Methyl-2-oxovaleric acid; Thiamine pyrophosphate; L-Valine; 2-Methylacetoacetyl-CoA; (S)-3-Hydroxyisobutyrate; Tiglyl-CoA; Butyryl-CoA; S-(2-Methylbutanoyl)-dihydrolipoamide; Alpha-ketoisovaleric acid; L-Isoleucine; R-Methylmalonyl-CoA; Methylmalonyl-CoA; Propionyl-CoA; (S)-Methylmalonic acid semialdehyde; (S)-b-aminoisobutyric acid; 2-Methyl-3-hydroxybutyryl-CoA; (S)-3-Hydroxyisobutyryl-CoA; Methacrylyl-CoA; (S)-2-Methylbutanoyl-CoA; S-(2-Methylpropionyl)-dihydrolipoamide-E; 4-Methyl-2-oxopentanoate; S-(3-Methylbutanoyl)-dihydrolipoamide-E; L-Leucine; 3-Methylglutaconyl-CoA; Succinyl-CoA; Methylmalonic acid |
| Pantothenate and CoA biosynthesis | Dephospho-CoA; Pantetheine 4'-phosphate; Pantetheine; 4-Phosphopantothenoylcysteine; D-Pantothenoyl-L-cysteine; D-4'-Phosphopantothenate; L-Cysteine; Pantothenic acid; Ureidopropionic acid; Dihydrouracil; L-Valine; Coenzyme A; Beta-Alanine; Uracil; Alpha-ketoisovaleric acid |
| beta-Alanine metabolism | Acrylyl-CoA; 3-Hydroxypropionyl-CoA; Hydroxypropionic acid; Malonyl-CoA; Beta-Alanine; L-Aspartic acid; Spermine; 1,3-Diaminopropane; 3-Aminopropionaldehyde; Ureidopropionic acid; Dihydrouracil; Anserine; Propionyl-CoA; Acetyl-CoA; Malonic semialdehyde; Spermidine; Uracil |
| Propanoate metabolism | (S)-Methylmalonic acid semialdehyde; Methylmalonyl-CoA; Propionyl-CoA; Propinol adenylate; R-Methylmalonyl-CoA; Succinic acid; Hydroxypropionic acid; Beta-Alanine; 2-Propyn-1-al; Acetyl-CoA; Malonyl-CoA; 2-Hydroxybutyric acid; Acrylyl-CoA; Propionic acid; Succinyl-CoA; 3-Hydroxypropionyl-CoA; Malonic semialdehyde; Propynoic acid; Acetoacetyl-CoA; 2-Ketobutyric acid |
| Butanoate metabolism | 3-Butyn-1-al; (R)-3-Hydroxybutyric acid; Acetoacetic acid; 3-Hydroxy-3-methylglutaryl-CoA; Acetyl-CoA; Acetoacetyl-CoA; (S)-3-Hydroxybutanoyl-CoA; Crotonoyl-CoA; Gamma-Aminobutyric acid; L-Glutamic acid; Butanoyl-CoA; Butanal; Succinic acid semialdehyde; Butyric acid; Oxoglutaric acid; Thiamine pyrophosphate; Pyruvic acid; 3-Butynoate; 1-Butanol; Succinic acid; 2-Hydroxyglutarate; 2-(a-Hydroxyethyl)thiamine diphosphate |
| Lysine degradation | L-Lysine; 4-Trimethylammoniobutanoic acid ; 4-Trimethylammoniobutanal; N6,N6,N6-Trimethyl-L-lysine; Protein lysine; Protein N6-methyl-L-lysine; Protein N6,N6-dimethyl-L-lysine; Crotonoyl-CoA; (S)-3-Hydroxybutanoyl-CoA; Saccharopine; Aminoadipic acid; Oxoadipic acid; Acetoacetyl-CoA; Glutaryl-CoA; Pipecolic acid; S-Glutaryldihydrolipoamide; Carnitine; 3-Hydroxy-N6,N6,N6-trimethyl-L-lysine; 5-Hydroxylysine; Protein N6,N6,N6-trimethyl-L-lysine; L-2-Aminoadipate 6-semialdehyde; Acetyl-CoA; (S)-2,3,4,5-Tetrahydropyridine-2-carboxylate |
| Glutathione metabolism | R-S-Cysteinylglycine; R-S-Glutathione; Glutathione; NADP; NADPH; Oxidized glutathione; Gamma-Glutamylcysteine; Glycine; L-Cysteine; L-Glutamic acid; Cysteinylglycine; Pyroglutamic acid; L-Amino acid; 5-L-Glutamyl-L-alanine; RX; Ornithine; Putrescine; Spermidine; Cadaverine; Tryparedoxin; Trypanothione; S-Substituted L-cysteine; Spermine; Aminopropylcadaverine; Tryparedoxin disulfide; Trypanothione disulfide |
| Purine metabolism | Guanosine diphosphate; Xanthine; D-Ribulose 5-phosphate; Phosphoribosyl pyrophosphate; L-Glutamine; 5-Phosphoribosylamine; Glycineamideribotide; Phosphoribosylformylglycineamidine; AICAR; SAICAR; 5-amino-1-(5-phospho-D-ribosyl)imidazole-4-carboxylate; RNA; Cyclic AMP; Adenosine triphosphate; dATP; ADP; dADP; Adenosine monophosphate; Adenylsuccinic acid; Inosinic acid; Adenosine; Deoxyadenosine monophosphate; Deoxyadenosine; Deoxyinosine; Xanthosine; IDP; Guanosine monophosphate; Xanthylic acid; Hypoxanthine; Inosine; Guanine; Deoxyguanosine; Allantoic acid; Uric acid; 5-Hydroxyisourate; Guanosine 3'-diphosphate 5'-triphosphate; Guanosine triphosphate; 2'-Deoxyguanosine 5'-monophosphate; dGDP; Guanosine; dGTP; Cyclic GMP; Sulfate; Adenosine phosphosulfate; Phosphoadenosine phosphosulfate; 5'-Phosphoribosyl-N-formylglycinamide; Inosine triphosphate; Xanthosine 5-triphosphate; Diadenosine tetraphosphate; P1,P4-Bis(5'-xanthosyl) tetraphosphate; Adenosine diphosphate ribose; Adenine; dIDP; 2'-Deoxyinosine triphosphate; Diadenosine triphosphate; Phosphoribosyl formamidocarboxamide; 5-Hydroxy-2-oxo-4-ureido-2,5-dihydro-1H-imidazole-5-carboxylate; 5-Aminoimidazole ribonucleotide; DNA; Ammonia; Urea; (S)-Ureidoglycolic acid; Guanosine 3',5'-bis(diphosphate); Adenosine 3',5'-diphosphate; Diguanosine tetraphosphate; dIMP; 5-Amino-4-imidazolecarboxyamide; (S)(+)-Allantoin |
| Aminoacyl-tRNA biosynthesis | L-Asparagine; tRNA(Asn); L-Histidine; tRNA(His); L-Phenylalanine; tRNA(Phe); L-Arginine; tRNA(Arg); L-Glutamine; tRNA(Gln); L-Cysteine; tRNA(Cys); Glycine; tRNA(Gly); tRNA(Asp) ; L-Aspartic acid; L-Serine; tRNA(Ser); L-Methionine; tRNA(Met); L-Valine; tRNA(Val); L-Alanine; tRNA(Ala); L-Lysine; tRNA(Lys); L-Isoleucine; tRNA(Ile); tRNA(Leu); L-Leucine; L-Threonine; tRNA(Thr); tRNA(Trp); L-Tryptophan; N10-Formyl-THF; L-Methionyl-tRNA; L-Tyrosine; tRNA(Tyr); L-Proline; tRNA(Pro); L-Glutamic acid; tRNA(Glu); Glutaminyl-tRNA; L-Asparaginyl-tRNA(Asn); tRNA(Sec); L-Seryl-tRNA(Sec); O-Phosphoseryl-tRNA(Sec); L-Histidyl-tRNA(His); L-Phenylalanyl-tRNA(Phe); L-Arginyl-tRNA(Arg); L-Cysteinyl-tRNA(Cys); Glycyl-tRNA(Gly); L-Aspartyl-tRNA(Asp); L-Seryl-tRNA(Ser); L-Valyl-tRNA(Val); L-Alanyl-tRNA; L-Lysyl-tRNA; L-Isoleucyl-tRNA(Ile); L-Leucyl-tRNA; L-Threonyl-tRNA(Thr); L-Tryptophanyl-tRNA(Trp); Tetrahydrofolic acid; N-Formylmethionyl-tRNA; L-Tyrosyl-tRNA(Tyr) ; L-Prolyl-tRNA(Pro); L-Glutamyl-tRNA(Glu); L-Glutamyl-tRNA(Gln); L-Aspartyl-tRNA(Asn); L-Selenocysteinyl-tRNA(Sec) |
| Glycerophospholipid metabolism | Phosphatidylethanolamine; Phosphatidylcholine; Dihydroxyacetone phosphate; LysoPC(18:1(9Z)); 1,2-Diacyl-sn-glycerol; Citicoline; Phosphorylcholine; Choline; Acetylcholine; O-Phosphoethanolamine; Ethanolamine; PA(16:0/16:0); Acyl-CoA; 1-Acyl-sn-glycerol 3-phosphate; CDP-diacylglycerol; Glycerol 3-phosphate; 1-Acyl-sn-glycero-3-phosphoethanolamine; 2-Acyl-sn-glycero-3-phosphoethanolamine; 2-Acyl-sn-glycero-3-phosphocholine; CDP-glycerol; PS(16:0/16:0); Phosphatidylglycerol; Dihydroxyacetone Phosphate Acyl Ester; CDP-Ethanolamine; 1-Phosphatidyl-D-myo-inositol; Glycerylphosphorylethanolamine; Glycerophosphocholine; Phosphatidyl-N-methylethanolamine; Phosphatidylglycerophosphate; Cardiolipin |
| Glycine, serine and threonine metabolism | L-Serine; Choline; Glyceric acid; Betaine; Guanidoacetic acid; 3-Phospho-D-glycerate; Dimethylglycine; L-Cystathionine; Glycine; Phosphoserine; Sarcosine; 5,10-Methylene-THF; L-Threonine; Lipoylprotein; Aminoacetone; Tetrahydrofolic acid; S-Aminomethyldihydrolipoylprotein; Dihydrolipoylprotein; D-Serine; Betaine aldehyde; Hydroxypyruvic acid; Creatine; Phosphohydroxypyruvic acid; L-Cysteine; Glyoxylic acid; L-2-Amino-3-oxobutanoic acid; Pyruvic acid; Carbon dioxide; 5-Aminolevulinic acid; Pyruvaldehyde; Ammonia |
| Steroid biosynthesis | Delta7-Avenasterol; Zymosterol intermediate 2; Calcidiol; 7-Dehydrocholesterol; Lathosterol; 5a-Cholest-8-en-3b-ol; 24-Methylenecholesterol; 5-Dehydroepisterol; Episterol; Cholesterol; 7-Dehydrodesmosterol; 4,4-Dimethyl-5a-cholesta-8,24-dien-3-b-ol; Avenasterol; Obtusifoliol; Delta 8,14 -Sterol; 5-Dehydroavenasterol; Farnesyl pyrophosphate; Presqualene diphosphate; (S)-2,3-Epoxysqualene; 4,4-Dimethylcholesta-8,14,24-trienol; 4a-Methylfecosterol; 4a-Carboxy-4b-methyl-5a-cholesta-8,24-dien-3b-ol; Lanosterin; 3-Keto-4-methylzymosterol; CE(16:1(9Z)); 5a-Cholesta-7,24-dien-3b-ol; Calcitriol; Campesterol; Desmosterol; 4,4-Dimethyl-5a-cholesta-8-en-3b-ol; Beta-Sitosterol; 24-Methylenelophenol; 24,25-Dihydrolanosterol; 4a-Methylzymosterol |
| Amino sugar and nucleotide sugar metabolism | N-Acetyl-D-glucosamine; N-Acetyl-D-Glucosamine 6-Phosphate; N-Acetyl-alpha-D-glucosamine 1-phosphate; Uridine diphosphate-N-acetylglucosamine; N-Acetylmannosamine; N-Acetylneuraminic acid 9-phosphate; N-Acetylneuraminic acid; Galactose 1-phosphate; Uridine diphosphate glucose; Glucose 1-phosphate; Uridine diphosphate glucuronic acid; Glucosamine 6-phosphate; Mannose 6-phosphate; Guanosine diphosphate mannose; Fructose 6-phosphate; Alpha-D-Glucose; D-Galactose; GDP-L-fucose; Fucose 1-phosphate; L-Fucose; N-Glycolylneuraminic acid; Cytidine monophosphate N-acetylneuraminic acid; Ferrocytochrome b5; Ferricytochrome b5; Chitobiose; Chitin; D-Mannose 1-phosphate; Glucose 6-phosphate; D-Mannose; beta-D-Fructose; Glucosamine; N-Acetyl-D-mannosamine 6-phosphate; Uridine diphosphategalactose; UDP-D-Xylose; Beta-D-Fructose 6-phosphate; GDP-4-Dehydro-6-deoxy-D-mannose; CMP-N-glycoloylneuraminate |
| Fatty acid metabolism | Palmityl-CoA; Hexanoyl-CoA; Butanoyl-CoA ; Acetyl-CoA; (S)-3-Hydroxybutanoyl-CoA; cis,cis-3,6-Dodecadienoyl-CoA; (S)-Hydroxyhexanoyl-CoA; (S)-Hydroxyoctanoyl-CoA; Octanoyl-CoA; (S)-Hydroxydecanoyl-CoA; Decanoyl-CoA (n-C10:0CoA); (S)-3-Hydroxydodecanoyl-CoA; Lauroyl-CoA; Primary alcohol; Glutaryl-CoA; (S)-3-Hydroxytetradecanoyl-CoA; Tetradecanoyl-CoA; Fatty acid; (S)-3-Hydroxyhexadecanoyl-CoA; Palmitic acid; (2E)-Hexadecenoyl-CoA; trans-2-Hexenoyl-CoA; Crotonoyl-CoA; Acetoacetyl-CoA; trans,cis-Lauro-2,6-dienoyl-CoA; Coenzyme A; 3-Oxohexanoyl-CoA; 3-Oxooctanoyl-CoA; (2E)-Octenoyl-CoA; 3-Oxodecanoyl-CoA; (2E)-Decenoyl-CoA; 3-Oxododecanoyl-CoA; (2E)-Dodecenoyl-CoA; Aldehyde; 3-Oxotetradecanoyl-CoA; (2E)-Tetradecenoyl-CoA; omega-Hydroxy fatty acid; 3-Oxohexadecanoyl-CoA; L-Palmitoylcarnitine |
| Primary bile acid biosynthesis | Cholesterol; Cholest-5-ene-3beta,26-diol; 25-Hydroxycholesterol; 7 alpha,26-Dihydroxy-4-cholesten-3-one; 4-Cholesten-7alpha,12alpha-diol-3-one; 7a-Hydroxy-cholestene-3-one; 5-b-Cholestane-3a ,7a ,12a-triol; (25R)-3alpha,7alpha,12alpha-Trihydroxy-5beta-cholestan-26-oyl-CoA; (25S)-3alpha,7alpha,12alpha-Trihydroxy-5beta-cholestan-26-oyl-CoA; 3a,7a,12a-Trihydroxy-5b-cholest-24-enoyl-CoA; 3a,7a,12a-Trihydroxy-5b-24-oxocholestanoyl-CoA; 3a,7a-Dihydroxy-5b-24-oxocholestanoyl-CoA; Chenodeoxycholoyl-CoA; Glycine; Taurine; 3alpha,7alpha,12alpha,26-Tetrahydroxy-5beta-cholestane; 3a,7a,12a-Trihydroxy-5b-cholestan-26-al; 3 beta,7 alpha-Dihydroxy-5-cholestenoate; 7-a,27-dihydroxycholesterol; 7a-Hydroxycholesterol; 7-a,25-Dihydroxycholesterol; Choloyl-CoA; 24-Hydroxycholesterol; 3alpha,7alpha-Dihydroxy-5beta-cholestanate; 3a,7a-Dihydroxy-5b-cholestane; 3 alpha,7 alpha,26-Trihydroxy-5beta-cholestane; 3a,7a-Dihydroxy-5b-cholestan-26-al; (25R)-3alpha,7alpha-Dihydroxy-5beta-cholestanoyl-CoA ; (25S)-3alpha,7alpha-Dihydroxy-5beta-cholestanoyl-CoA; 3a,7a-Dihydroxy-5b-cholest-24-enoyl-CoA; 3a,7a,12a-Trihydroxy-5b-cholestanoic acid; 3a,7a,12a-Trihydroxy-5b-cholestanoyl-CoA; 3a,7a,12a,24-Tetrahydroxy-5b-cholestanoyl-CoA; 3 beta-Hydroxy-5-cholestenoate; (24S)-Cholest-5-ene-3beta,7alpha,24-triol; 7 alpha-Hydroxy-3-oxo-4-cholestenoate; 7a,12a-Dihydroxy-5b-cholestan-3-one; 7a-Hydroxy-5b-cholestan-3-one; Chenodeoxycholic acid; Chenodeoxycholic acid glycine conjugate; Taurochenodesoxycholic acid; 7alpha,25-Dihydroxy-4-cholesten-3-one; Cholic acid; Glycocholic acid; Taurocholic acid; 7 alpha,24-Dihydroxy-4-cholesten-3-one |
